# Navigating the complex landscape of benzodiazepine- and Z-drug diversity: insights from comprehensive FDA adverse event reporting system analysis and beyond

**DOI:** 10.3389/fpsyt.2023.1188101

**Published:** 2023-06-23

**Authors:** Filip Koniuszewski, Florian D. Vogel, Irena Dajić, Thomas Seidel, Markus Kunze, Matthäus Willeit, Margot Ernst

**Affiliations:** ^1^Department of Pathobiology of the Nervous System, Center for Brain Research, Medical University Vienna, Vienna, Austria; ^2^Department of Psychiatry and Psychotherapy, Medical University of Vienna, Vienna, Austria; ^3^Department of Pharmaceutical Sciences, University of Vienna, Vienna, Austria

**Keywords:** Z-drugs, benzodiazepine binding sites, sex differences, adverse events, pharmacovigilance, side effects, FDA adverse event reporting system, benzodiazepine

## Abstract

**Introduction:**

Medications which target benzodiazepine (BZD) binding sites of GABAA receptors (GABAARs) have been in widespread use since the nineteen-sixties. They carry labels as anxiolytics, hypnotics or antiepileptics. All benzodiazepines and several nonbenzodiazepine Z-drugs share high affinity binding sites on certain subtypes of GABAA receptors, from which they can be displaced by the clinically used antagonist flumazenil. Additional binding sites exist and overlap in part with sites used by some general anaesthetics and barbiturates. Despite substantial preclinical efforts, it remains unclear which receptor subtypes and ligand features mediate individual drug effects. There is a paucity of literature comparing clinically observed adverse effect liabilities across substances in methodologically coherent ways.

**Methods:**

In order to examine heterogeneity in clinical outcome, we screened the publicly available U.S. FDA adverse event reporting system (FAERS) database for reports of individual compounds and analyzed them for each sex individually with the use of disproportionality analysis. The complementary use of physico-chemical descriptors provides a molecular basis for the analysis of clinical observations of wanted and unwanted drug effects.

**Results and Discussion:**

We found a multifaceted FAERS picture, and suggest that more thorough clinical and pharmacoepidemiologic investigations of the heterogenous side effect profiles for benzodiazepines and Z-drugs are needed. This may lead to more differentiated safety profiles and prescription practice for particular compounds, which in turn could potentially ease side effect burden in everyday clinical practice considerably. From both preclinical literature and pharmacovigilance data, there is converging evidence that this very large class of psychoactive molecules displays a broad range of distinctive unwanted effect profiles - too broad to be explained by the four canonical, so-called “diazepam-sensitive high-affinity interaction sites”. The substance-specific signatures of compound effects may partly be mediated by phenomena such as occupancy of additional binding sites, and/or synergistic interactions with endogenous substances like steroids and endocannabinoids. These in turn drive the wanted and unwanted effects and sex differences of individual compounds.

## 1. Introduction

### 1.1. GABAA receptors

GABAA receptors are a heterogeneous protein family in the nervous system and in non-neuronal tissues. They assemble as transmembrane homo-or heteropentameric anion channels, which specifically conduct bicarbonate and chloride anions and are gated by the endogenous ligand GABA. In most instances, opening of neuronal channels facilitates chloride movement from the extracellular space into the cytoplasm, with a net inhibitory effect (1–3). GABAARs can be categorized into (1) postsynaptic receptors, which facilitate fast point to point communications between cells following action potentials, (2) extrasynaptic ones, which show high GABA affinity and a steady, non-desensitizing stream of ionic flow in order to provide tonic inhibition, as well as (3) perisynaptic receptors thought to chiefly gate synapses (4). Moreover, presynaptic GABAA receptors were described (5–7).

Due to the existence of 19 GABAA receptor genes encoding for α1-6, β1-3, γ1-3, δ, ε, π, ρ1-3, θ subunits in human and non-human mammals, and variants from splicing and RNA editing, the number of possible GABAAR pentamers is vast even considering the hitherto identified assembly rules (8–16). It is generally believed that most receptors contain two to three β- (or β-like subunits), one or two α-subunits, and one odd subunit which is most commonly γ2, oriented in a counter-clockwise manner, in α-β-α-γ-β-order. However, there is still an overwhelming number of receptor subtypes with unknown or divergent native receptor composition, assembly and stoichiometry (17, 18). Their physiological functions and pharmacological properties vary greatly, as known from heterologous expression systems as well as *in vitro* and *in vivo* studies in rodent systems (19–24).

### 1.2. Benzodiazepines and Z-drugs

**Figure 1:**
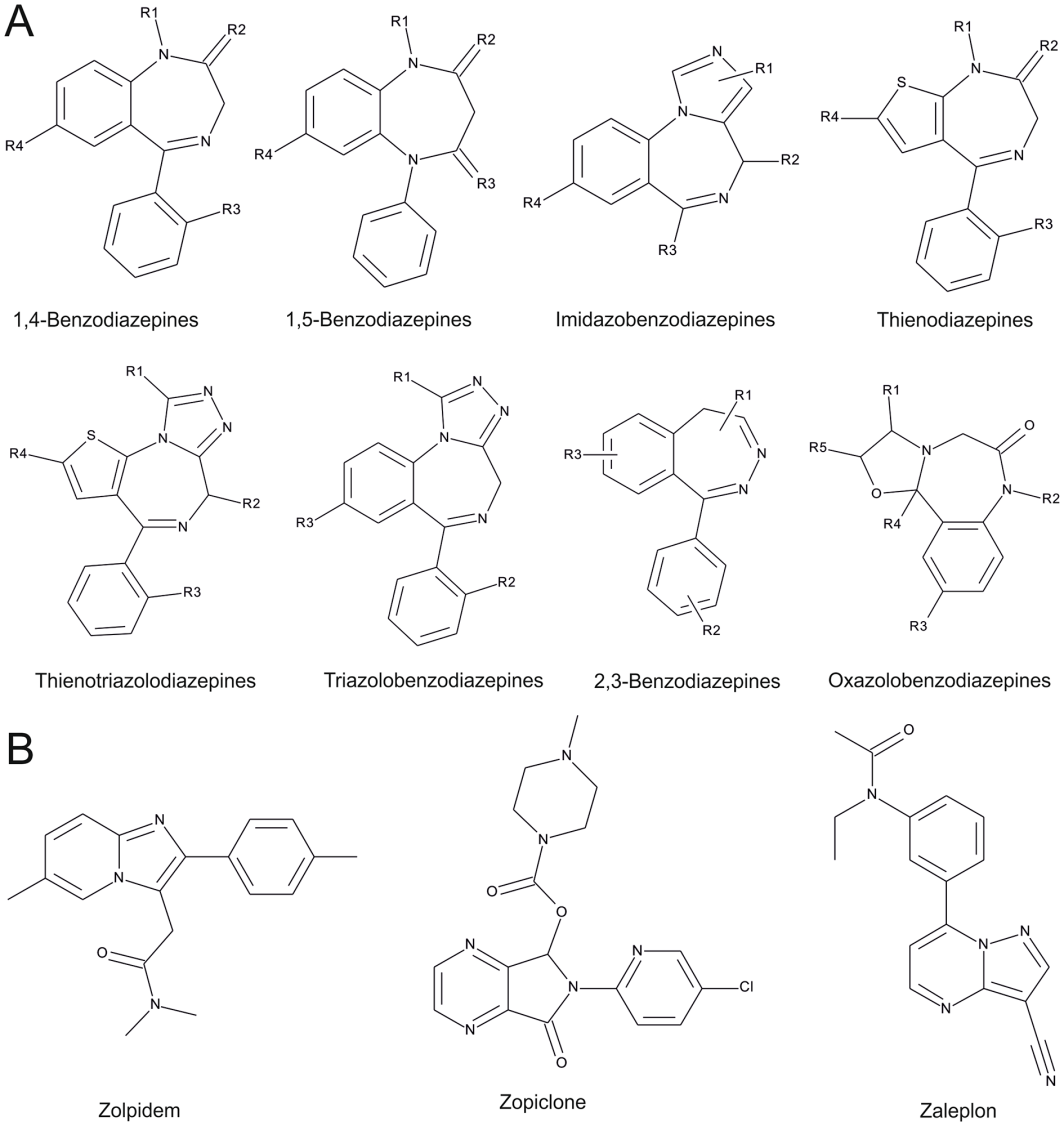
Chemical entities of benzodiazepines and Z-drugs, as in “New benzodiazepines in Europe review 2021” (26). **(A)** Benzodiazepine scaffolds are depicted. **(B)** Z-drug scaffolds, note that zopiclone comprises two entities (enantiomers), which are not reflected in this 2D- representation.

Benzodiazepines have dominated the pharmaceutical market of GABAA receptor targeting compounds since their introduction in the 1960s by Hoffmann La Roche (25). At the time, they replaced the previous generation of GABAA receptor targeting central nervous system (CNS) depressants, the barbiturates, due to a better pharmacological profile and safer use. They are a heterocyclic class of molecules chemically defined by an aromatic benzyl ring annulated to an unsaturated diazepine-ring (Figure 1). The compounds that incorporate a 1,4 – diazepine partial structure are the ones most frequently used clinically. To exert effects at low doses, BZDs require a high affinity binding site on GABAA receptors which is known to be localized at extracellular interfaces between an α1-3,5 “principal” subunit, together with a γ1-3 “complementary” subunit (27–33). Preclincial research and the low abundance of the γ3 subunit have led to the notion that the four sites formed by α1-3,5 together with γ2 account for the major share of drug effects that are mediated by the resulting four high affinity binding sites. Many receptors that lack the high affinity sites still can be modulated by BZDs in higher (micromolar) concentrations but lack low concentration BZD effects (34–36). However, it should be noted that the distinction is largely based on data originating from heterologous expression systems which do not account for endogenous GABAA receptor modulators and their allosteric interactions with BZD effects (37, 38).

As a class, BZDs have a broad variety of therapeutic effects, including anxiolysis, hypnosis, sedation, muscle relaxation and anticonvulsant effects (39–42). Dose-dependent euphorogenic and amnestic actions are described as well, which might contribute to their popularity in recreational and illicit use (43). In the Anatomical Therapeutic Chemical Classification (ATC) System, BZDs run under the codes N03 (antiepileptics), and N05 (psycholeptics) in which they are further divided into N05B (anxiolytics) and N05C (hypnotics and sedatives). They are useful therapeutics in many diseases and disorders, such as anxiety disorders, epilepsy or sleeping disorders. However, in most therapeutic regimens their broad pharmacological profile evokes unwanted or adverse effects in addition to the wanted effects. General side effects of BZDs include cognitive impairment (44), increased risk of fall and injury in the elderly (45), disturbance of sleep architecture (46), sedation, and muscle relaxation, among others (42, 47, 48). Sudden discontinuation after prolonged use may lead to withdrawal symptoms such as depressive mood, irritability, sleep disturbances, muscular tension, and tremor or even grand mal-like seizures. Treatment-emergent BZD use disorder is a rare, but sometimes serious adverse drug reaction. Additionally, in less than 1 % of patients or users, BZDs can induce paradoxical reactions ranging from talkativeness, restlessness, hyperactivity, excessive movement, to agitation and aggressive behavior in word and action, or even to seizures (46, 49, 50). Juveniles and elderly are especially susceptible to adverse effects (39).

Another group of molecules which target the high affinity benzodiazepine binding sites on GABAARs, are the Z-drugs: zaleplon, zolpidem, zopiclone and eszopiclone (see Figure 1). They were introduced in the 1990-ies and marketed as drugs of the millennium with claims for lower abuse potential and fewer side effects compared to BZDs. However, since their launch, the number of adverse event reports connected to the Z-drugs has been rising. They were shown to produce euphorogenic effects like prominently abused benzodiazepines such as lorazepam (Ativan), alprazolam (Xanax) and flunitrazepam (Rohypnol) (51). In addition, paradoxical reactions similar to those of BZDs have been described for Z-drugs as well (52). Overall, the side effects for Z-drugs are converging toward the ones observed for BZD administration, apart from a better performance on some cognitive measures in older populations (53). We will refer here to benzodiazepines and Z-drugs together as “BZ-site ligands” for brevity.

A variety of prescription drugs can affect benzodiazepine pharmacokinetics and effects by interfering with their liver metabolism through the cytochrome P450 (CYP) system, especially isoenzyme 3A4 and 2C19 action (54–57). This can lead to accumulation of the compounds, with severe side effects, or to therapy failure due to accelerated clearance of BZ-site ligands. Combining diazepam (Valium) with drugs such as rifampicin or the antiepileptic drug carbamazepine can dramatically accelerate its clearance (58–60). Hormonal oral contraceptives, on the other hand, can reduce clearance and increase the half-lifetime for multiple benzodiazepines (61–65). Natural grapefruit juice can severely impair diazepam metabolism by inhibition of CYP3A4 (66, 67) leading to clinically relevant stronger diazepam effects and accumulation. It has been observed that additional benzodiazepines can interfere with other CYP isoenzyme activity and/or with glucuronidation (68–71).

### 1.3. Useful or problematic drugs: controversial issues concerning dose escalation, non-medical use, and unwanted effect severity

Between 1996 and 2014 the number of adults in the US that filled prescriptions for BZ-site ligands increased significantly (8.1 million, 4.1% to 13.5 million; 5.6%) (72). Accordingly, the total filled quantity tripled and overdose deaths involving BZ-site ligands quadrupled from 0.58 to 3.07 per 100.000 adults. There is an evident gap between prescription rates of BZ-site ligands between sexes reported in multiple sources, such that women receive prescriptions for these drugs about twice as often as men (73, 74). Remarkably, despite this fact and their widespread usage in the clinics, coherent systematic studies on sex differences in effects of BZ-site ligands are rare. The existing studies point toward a controversy in terms of substance misuse risk due to sex with some indicating male sex as a risk factor (75–77) and others vice versa (78–80). However, due to different study designs, comparison among them is difficult.

Owing to their widespread *in vivo* effects, BZ-site ligands are prominent among commonly misused drugs. Since they have mainly CNS depressant effects, they are categorized as “downers” (81). In the US, all benzodiazepines are controlled in schedule IV of the “Controlled Substances Act” meaning they are considered to have relatively low addictive properties while serving a medical need. Nonetheless, BZDs and to a similar extent Z-drugs can cause physical and psychological dependence after relatively short periods of time, which is why the rule for treatment regimen is “as short as possible, as long as needed.” An added concern is that BZ-site ligands may induce drug tolerance in many of their effects, meaning that a higher dose is required for achieving the same effects.

Therefore, although they are generally perceived as a safe class of compounds, BZDs and Z-drugs can be problematic in long term treatments and illicit drug use. If taken alone, the potential of benzodiazepine overdose to cause fatal adverse effects is comparatively low in contrast to other depressants, such as barbiturates, but existent (82–84). Between 2005 and 2011 the emergency department visits that involved BZ-site ligands almost doubled, according to the DAWN (Drug Abuse Warning Network) report (84). The risk for serious outcomes during an emergency department visit was higher for benzodiazepine users compared to non-users, and was escalated further by combining benzodiazepines with alcohol or opioids (84). In addition, BZDs have been shown to approximately double the risk for motor vehicle accidents (85), and similar effects have been described for zopiclone (86, 87).

BZ-site ligands are often not a primary drug of abuse, but are taken in combination with other drugs (43). In particular, BZDs with a rapid onset of action can create euphoric effects, usually observed at higher concentrations. Diazepam (Valium) and alprazolam (Xanax) are combined with methadone to potentiate its mood enhancing effect further (43). Cocaine and other stimulant users utilize BZDs to mitigate side effects (43) or for “coming down.” The analysis of more than 1,200 oxycodone related drug abuse deaths from a postmortem database highlighted the prevalence of diazepam co-abuse in oxycodone users (84), as also described in other sources (88). The combination of alcohol and BZDs is particularly problematic given the low inhibition threshold of alcohol procurement by its social acceptance and easy accessibility (89). Furthermore, alcohol and BZ-site ligands both chiefly act as depressants, thus exerting a compounded effect when taken together. There is some evidence that for individuals with alcohol use disorder (AUD) a stronger psychoactive effect can be achieved after benzodiazepine administration. People with AUD in their familial history may also experience a different sensitivity and effects of alprazolam (90–93). Studies exist which describe drug – alcohol interactions and adverse outcomes that are associated with BZ-site ligands, but systematic comparisons between individual drugs are lacking. Thus, it remains unclear for most approved substances whether they are more or less problematic in different forms of medical and non-medical use, despite considerable anecdotal evidence that suggests that specific compounds are particularly well suited, e.g., as date rape drug, or have tendencies to elicit bad trips.

After all, Bz-site ligands have been an indispensable part of everyday clinical practice for decades and they remain so today (94). Attempts to restrict their use via tighter regulatory requirements for their prescription were followed by an increase in overdose emergencies involving drugs with a less favorable safety profile (95–97). Moreover, if due to excessive Bz-site ligand doses, acute sedation or respiratory depression are readily antagonized by intravenous flumazenil in clinical or emergency medicine settings. Thus, increased awareness and a more detailed understanding of the mechanisms mediating unwanted and at times dangerous BZD effects, in addition to supporting rational clinical decision making, could help to promote developing drugs with similar benefits but even more favorable risk profiles.

### 1.4. FAERS and use of pharmacovigilance data

At least partly because those substances are no longer protected by patents, comprehensive controlled studies and interindividual substance comparisons according to current scientific standards are lacking for the majority of BDZs & Z-drugs among indications in which they are currently used.

Pharmacovigilance is a rapidly growing scientific discipline that strives to detect, assess, understand and prevent drug-related issues and adverse events, thus in short includes every activity that is connected to better drug safety (98). To collect real world post marketing drug-adverse event observations, the U.S. government provides a federal database called FDA Adverse event reporting system (FAERS). The FAERS database includes adverse events, medication errors and product quality complaints that were submitted to the FDA by either health care professionals such as prescribers or pharmacists, but also by patients or other public members (99). Pharmacovigilance analysis typically utilizes these metrics to determine if a drug is associated with an adverse event. A greater value for these measures signifies a more substantial association between the medication and the unfavorable outcome. Thus, post-marketing pharmacovigilance data such as those in FAERS can provide highly useful signals for adverse reactions that were not observed in the early phases of the drug approval procedure. Due to the inherent limitations of the real-world data, such as a gap in provided dosages of the reported drug, co-usage of other substances, a lack of demographic data and others, specialized methods of data analysis have been developed (100–102). It is generally understood that a strong signal implies an association between a drug and an outcome, but cannot provide any evidence for causation. Thus, and due to other properties of real-life observations, pharmacovigilance data is not suitable for comparative pharmacology (101). However, it is the only data available to generate hypotheses on the basis of large numbers of real world observations and across a substantial number of drugs.

**Figure 2:**
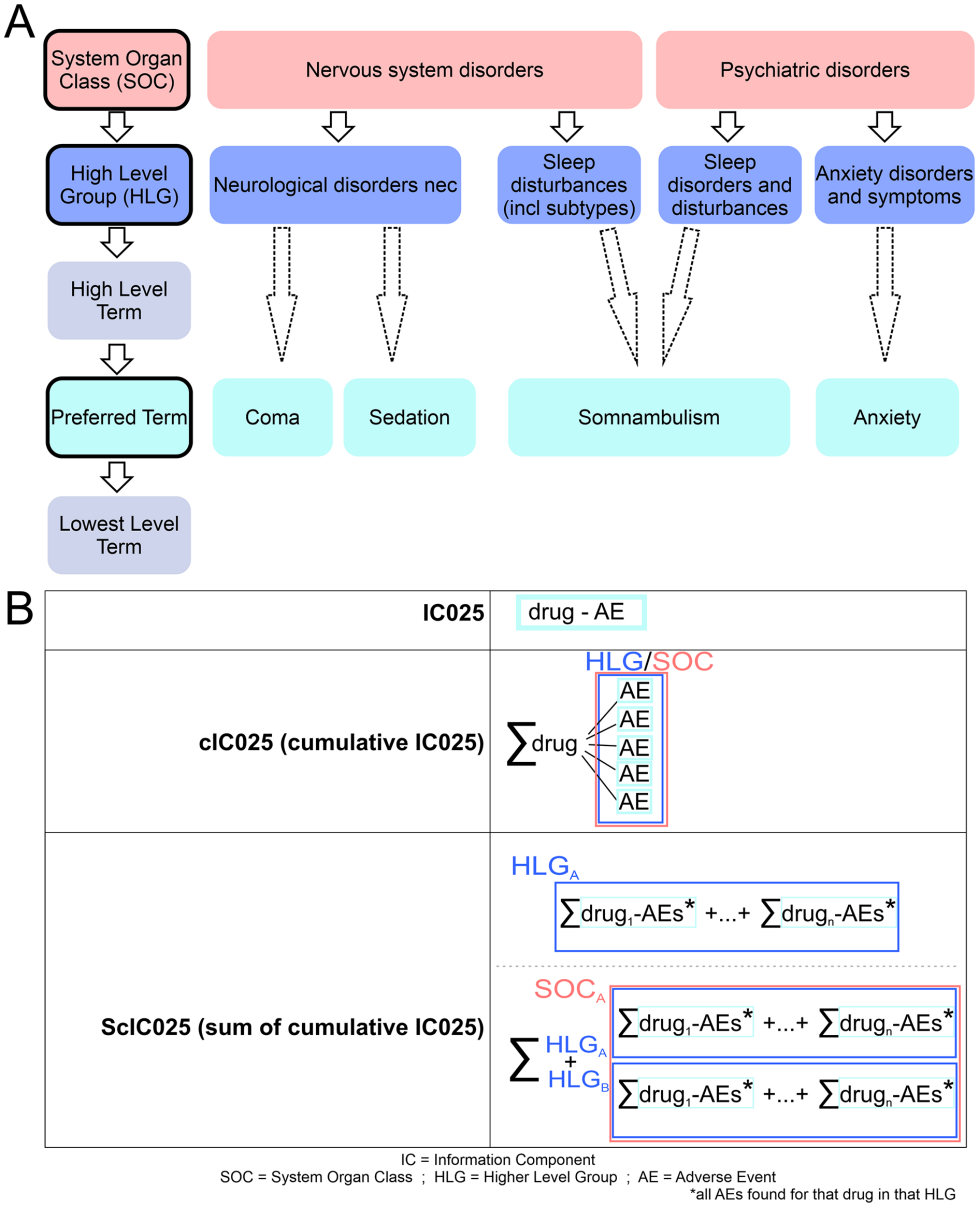
Overview of MedDra dictionary system and employed IC025 usage. **(A)** The five levels of MedDra hierarchy are displayed with specific examples to them; red: system organ class (SOC); blue: High level group (HLG), Cyan: Preferred term (PT). Arrows indicate the direction from higher levels to lower levels, dashed arrows indicate that we surpass the high level terms in the analysis shown in this work. **(B)** Different IC025 values and their respective calculations are shown. IC025 reflects on the drug-AE association; cIC025 is the sum of all IC025s for a drug within an HLG; ScIC025 gives the summation of cumulative IC025s for a HLG or SOC.

FAERS encourages use of “preferred terms” to report adverse events in MedDRA terms (See Figure 2). The MedDRA dictionary hierarchy is a categorization of medical terminology, which hence allows to analyze FAERS reports at the different MedDRA levels. The five levels of the dictionary are System Organ Class (SOC), High Level Group Term (HLGT), High Level Term (HLT), Preferred Term (PT), and Lowest Level Term (LLT) (103). For an overview of the MedDRA hierarchy and which levels were used in this study, see Figure 2A.

Since Bz-site ligands (comprising benzodiazepines and Z-drugs) are a broadly prescribed class of medications, the number of reports connected to their usage is vast. However, no comprehensive comparison between reports of individual compounds for Bz-site ligands has been performed to our knowledge yet. Here, we employ disproportionality analysis, which provides mathematically well-defined parameters for the strength of an association signal (104). Specifically, the commonly used information component (IC) value gives a measure of the strength of the quantitative dependency between the specific drug and the reported adverse event. Here we use the IC025, see Figure 2 and methods, which defines the endpoint of the 95% credibility interval (100). We analyzed a large FAERS dataset in order to generate individual drug profiles. We found and reported tendencies of drug-heterogeneity, some of which are confirmed by other sources containing clinical study data.

Molecular foundation for drug heterogeneity may be triggered by a variety of off-target and on-target effects. Conducting systematic investigations to explore every potential off-target effect of a drug may be impractical, given the vast number of molecules in the body. Thus, off target effects were not further considered in this study. For on-target heterogeneity, structural data provides hypotheses for mechanisms that can drive a multiplicity of overlapping and non-overlapping effects of the investigated drugs. These largely stem from multiple binding sites and their cooperativity at various receptor subtypes. The compounds for which informative FAERS records exist were thus also examined in terms of their chemical features that drive the pharmacodynamics with the hope to identify common drug properties that drive certain unwanted effects and a short overview of binding site heterogeneity within the family of GABAARs is also provided.

## 2. Results

**Figure 3:**
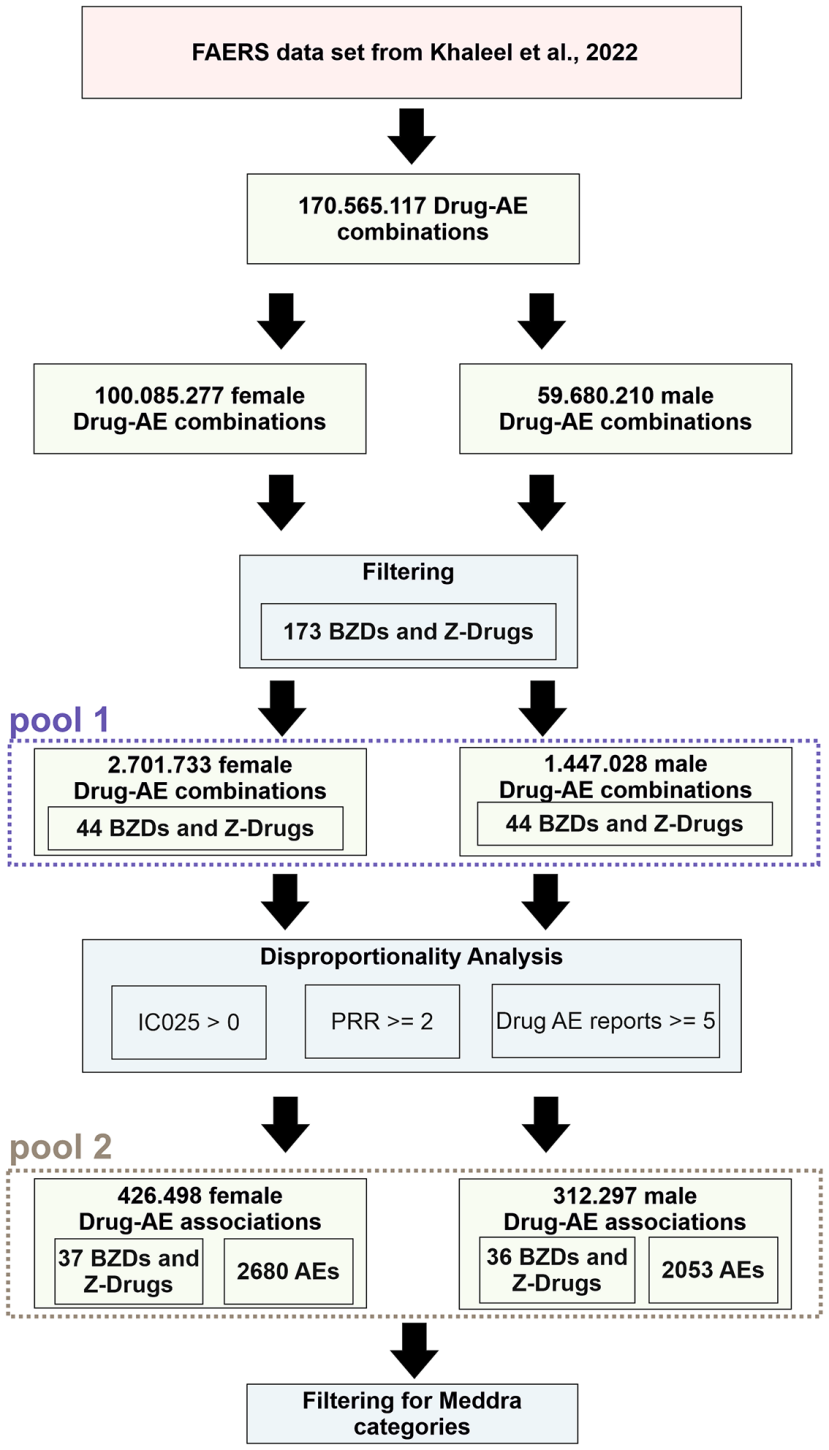
Pipeline that was performed on the FAERS dataset. Green boxes display the results obtained after the filtering steps, which are represented by blue boxes. Data that was used for analysis according to MedDRA categories is identified as “pool 1” and “pool 2” respectively.

We mined the publicly available FAERS (FDA Adverse Event Reporting System) data set from Khaleel et al. (105) to establish pharmacovigilance profiles per drug and sex, for all Bz-site ligands with sufficient data. For all steps of our analysis, the datasets from female and male reports were treated separately to obtain individual results per sex, in the same vein as done by Drug Central (106). The applied workflow is displayed in Figure 3 (see also the Methods section). The full data set comprised 170.565.117 drug – adverse event combinations, including 100.085.277 female drug-adverse event combinations and 59.680.210 male drug-adverse event combinations. These reports were filtered for 173 drugs composed of benzodiazepines and Z-drugs from our drug list (for detailed information, see Methods section and Supplementary Item 1). The filtering process left us with 2.701.733 female drug-adverse event combinations and 1.447.028 male drug-adverse event combinations, from the use of 44 benzodiazepines, for which a FAERS entry exists, and which are referred to as data pool 1 for brevity (see Figure 3; Supplementary Table S1). Disproportionality analysis was performed to identify drug-adverse event associations. Our primary criteria for inclusion of a record into data pool 2 were the commonly used thresholds of PRR > 2 and IC025 > 0 (107–110) as well as the existence of five or more records. Supplementary Item 2 provides the composition of pools 1 and 2 (see Figure 3) with respect to the total reports per drug that were analyzed. The main criterion used for subsequent data filtering was the IC025 value, as suggested by the UMC (Uppsala Monitoring Center) (111), since higher IC025 values reflect a stronger signal. Only 39 of the 44 drugs found in the dataset met the applied criteria and thus were used for further analysis (pool 2). The raw data is provided in Supplementary Items 3, 4 in Excel format.

The records from pool 2 (after the disproportionality analysis) were analyzed with the use of the MedDRA categories, and in some instances pool 1 data was utilized for comparison. To analyze pool 2 data, which contains only drug-AE associations, we employ the following nomenclature (see Figure 2B): IC025 denotes the value for a particular drug and an individual adverse effect combination where usually only positive values from pool 1 were used in the downstream calculations of aggregate values. Simple sums, cumulative IC025 (cIC025), are the aggregate of all positive IC025 values for a specific drug within a category (HLG or SOC). Summative cumulative IC025 (ScIC025) indicates the sum of cIC025-values for all drugs combined within the group (HLG or SOC), see Figure 2B.

### 2.1. Overview across all SOCs

**Figure 4:**
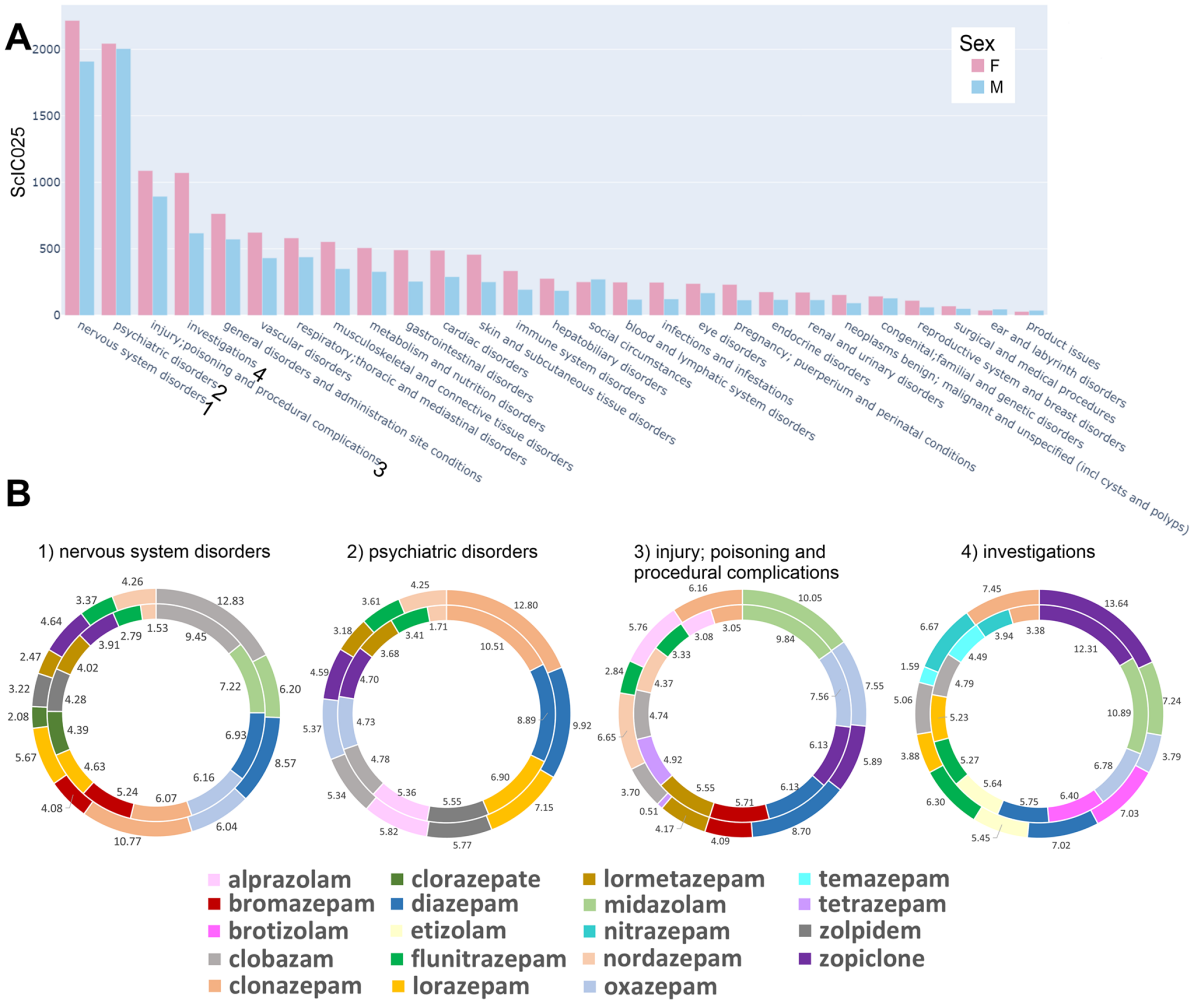
Distribution of AE associations across all organ system classes: **(A)** Summed cumulative IC025 (ScIC025) values per SOCs for all drugs which had a positive IC025 separated by sex. Bars reflecting female reports are light red, those reflecting male reports are light blue. **(B)** Pie charts are presented for the top 10 drugs for each sex (together 10 or more) with the highest cIC025 contribution to the four highest ranked system organ classes (SOCs) in terms of summed cIC025 (ScI025). The size of the displayed segments corresponds to the cIC025 contribution of each drug to the summed cumulative IC025 (ScIC025) and is shown as percentage. The outer circles reflect data for males, while the inner circles represent data for females, with the drugs sorted according to the female ScI025 rank values, starting at the top and descending in clockwise direction.

At the highest MedDRA level of system organ classes (SOCs), associations were obtained for all 39 drugs, in 27 SOCs, Figure 3. To obtain an overview, the summed cumulative IC025 (ScIC025) values per SOC were computed and are displayed in Figure 4A. Not surprisingly, the largest summed cumulative signals were observed for “nervous system disorders” and “psychiatric disorders,” together comprising the “neuropsychiatric” group. Owing to the widespread non-medical use of Bz-site ligands, it is not unexpected that the SOC “injury, poisoning and procedural complications” also displays a high ScIC025 as seen in Figure 4A, closely followed by “investigations.” The top four SOCs were fully decomposed into the contributing HLGs, see Supplementary Figures S1–S3.

For each of the four top SOCs, we ranked the contributing drugs by ScIC025 over the whole SOC to investigate the gross contributions. The top 10+ drugs for each SOC are depicted in Figure 4B, where more than 10 drugs are shown because the top 10 differed between the sexes. It is noteworthy that each SOC features a unique drug ranking, and the top ranked drug is different for all four analyzed SOCs. Clobazam is top ranked in “nervous system disorders” for both sexes, and occurs in the top 10 for the other three SOCs as well. In the “psychiatric disorders,” the top ranked drug is clonazepam, which is also found among the top 10 in all four datasets. Its relative contribution to each SOC differs in part considerably between sexes. Midazolam, as a procedural anesthetic, is the top ranked compound in the HLG “injury, poisoning and procedural complications,” and is not among the top 10 in the “psychiatric disorders.” The SOC “investigations” is very heterogeneous, as it does not reflect a single organ system but comprises parameter changes across all SOCs. There, striking differences in cIC025 between the male and female signals occur for several drugs, e.g., temazepam and nitrazepam. Closer inspection of this SOC and its constituent subgroups, see Supplementary Figure S3, reveals high ScIC025 value for females in cardiac investigations. This is also matched by the higher ScIC025 for females in the SOC “cardiac disorders,” Figure 4A.

**Figure 5:**
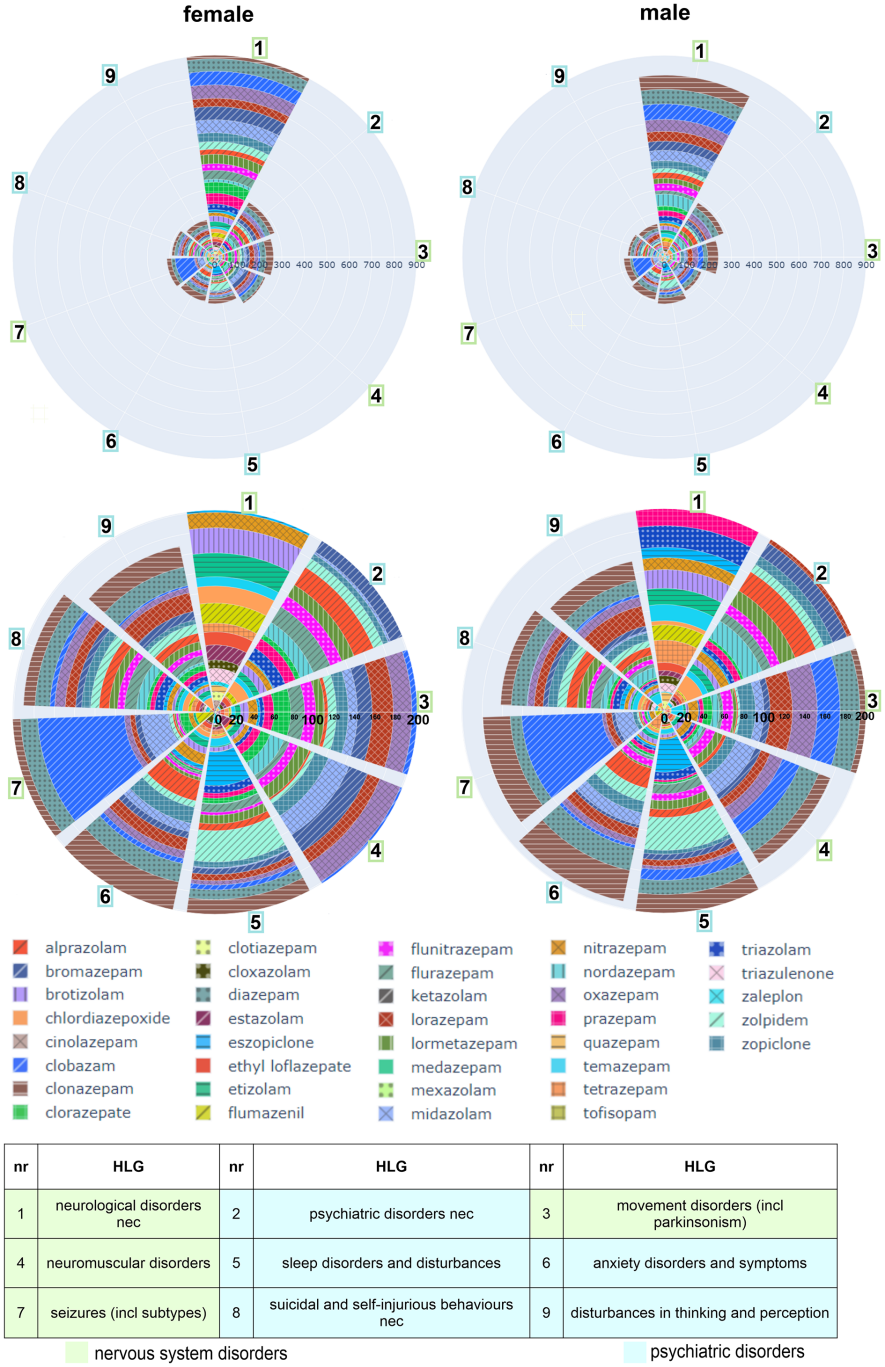
Distribution of AE associations across the organ system classes “nervous system disorders” and “psychiatric disorders”: Higher level groups from the “nervous system -” and “psychiatric – disorders,” in which a summed cumulative IC025 of both sexes adds up to 300 or higher, are displayed as polar bar charts. The individual cIC025 contribution per compound to the summed one in the neuro-psychiatric disorders is displayed as patterned segments as defined in the legend. Drugs in the polar bar chart are sorted by their total contribution of ScIC025 within the whole SOC per sex from high to low, with the higher ranked drugs at the outer rims. The table below identifies the nine largest contributions from the top two SOCs. green: nervous system disorders; blue: psychiatric disorders.

Results obtained for the four analyzed SOCs suggest a heterogeneous side effect pattern associated with individual compounds – while some drugs occur only in the top 10 of individual SOCs (e.g., brotizolam only occurs in “investigations”), others dominate several or all of the SOCs. In order to investigate drug heterogeneity upon more detailed decomposition, we zoomed further into the top two SOCs – after merging them into “neuropsychiatric reports,” see Figure 5. Supplementary Figures S1, S2 provide more details on the SOCs “nervous system disorders” and “psychiatric disorders” separately.

### 2.2. Analysis of neuropsychiatric AEs

Closeup analysis of the neuropsychiatric SOCs was performed in a next step. The largest contributing higher level groups in the neuropsychiatric SOCs are displayed in Figure 5. They comprise groups with “neurological/psychiatric disorders not elsewhere classifiable (nec),” two groups with disturbances in movement/motor systems, signs and symptoms related to sleep, anxiety signs, seizures, suicidal and self-injurious behaviors, and a group with disturbances in thinking and perception. The ScIC025 per HLG differs only to a small degree between sexes (Figure 5). As a next step we looked a drugs’ contribution in terms of cIC025 to each HLG.

In line with the large contributions to the ScIC025 by clobazam, clonazepam and diazepam to the whole nervous system and psychiatric SOCs, these drugs are seen to have rather large cIC025 values in the individual neuropsychiatric HLGs as well. However, heterogeneity emerges at this level too: Clobazam is seen to contribute with a considerable association to the “seizures” HLG, as we have noted previously (112) but, e.g., with only a small signal to “anxiety disorders and symptoms.” In the two groups concerned with movement and muscle symptoms, several drugs carry different association strength as can be seen in Figure 5B where clobazam has a stronger signal in males.

### 2.3. Neurological disorders not elsewhere classified

**Figure 6:**
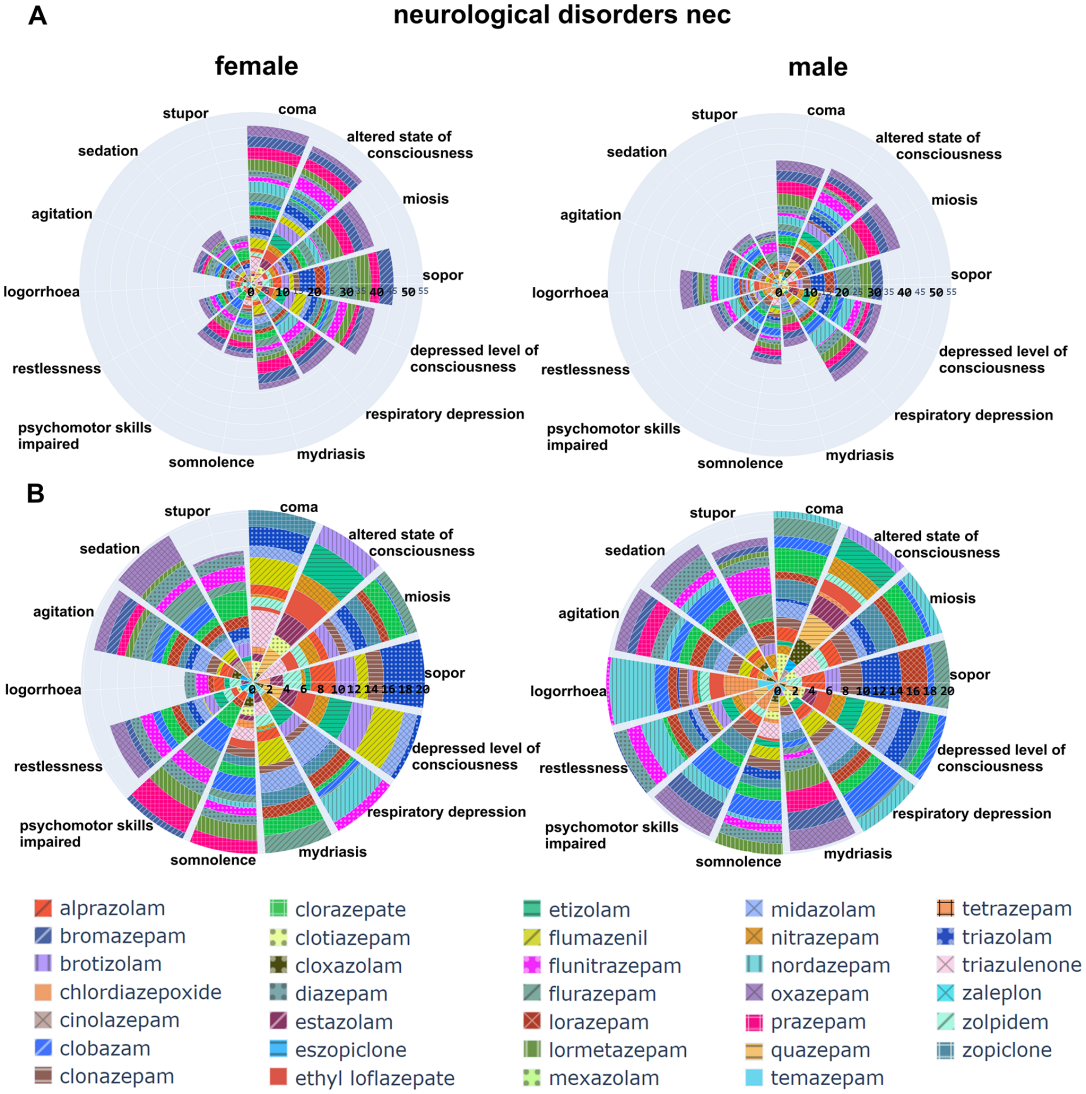
Distribution of AE associations across the HLG “neurological disorders nec”: The top two polar bar charts display the individual adverse events that have a signal in this HLG as cumulative IC025 across all contributing drugs. For plotting, a cutoff was used: all AEs with a cumulative IC025 > 30 for both sexes added are plotted, the full dataset is in Supplementary Items 3, 4. The drugs are identified in the list on the bottom of the graph. Panel **B** is an enlarged view of panel **A**, note the cIC025 scale (0–20).

Interestingly, the HLG 1 (“neurological disorders nec,” Figure 5A) accounts for almost half of the ScIC025 from the SOC “nervous system disorders” with a summed cumulative IC025 value of about 900 in females and 800 in males. This HLG was thus analyzed in detail at the level of the individual adverse event associations (= IC025 values), see Figure 6.

The major contributing AEs to this HLG are signs of sedation and over-sedation, including sedation, somnolence, sopor and coma, all indicative of CNS depression of various degrees (see Figure 6). The second largest group comprising agitation, restlessness and logorrhoea reflects paradoxical responses (see Figure 6). It is interesting to note that the cumulative signal for logorrhoea differs markedly between the sexes, and displays some drug specificity. For example, logorrhoea sticks out in the male dataset (IC025 = 5.3), and shows no association for females. This is due to the low number of reports (<5) and thus was not taken into pool 2. More detail can be found in the provided data in Supplementary Items 3, 4. In total, a large share of cIC025 in the neuropsychiatric groups thus reflects the known and expected signs of sedation and over-sedation on the one hand side, and paradoxical reactions on the other hand side.

### 2.4. Highest ranked psychiatric HLGs

**Figure 7:**
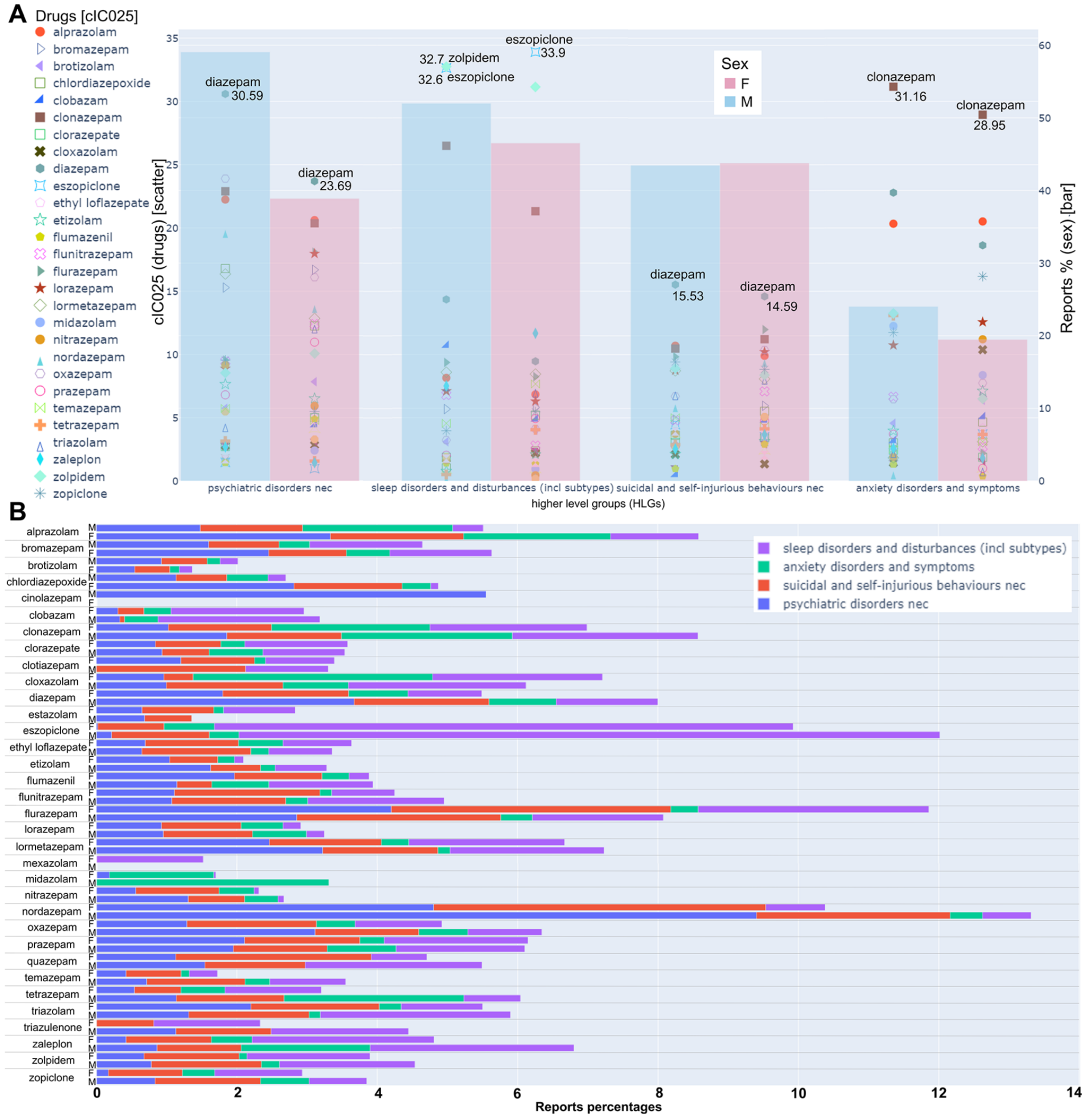
Detailed analysis of the top four psychiatric HLGs. **(A)** The left axis refers to the scatter plot. For each drug, the cumulative IC025 in the respective HLG is displayed. The drugs with the highest cIC025 value within each HLG are displayed on the graph; the respective cIC025 value is given next to the drug. The right axis refers to the bar graphs; blue: male, red: female; Bar height indicates the report percentage of all drugs within the HLG in relation to the reports of all drugs in all HLGs, as specified in the methods. Calculated as specified in the methods. **(B)** For each drug, the percentage of reports for a HLG in relation to the total reports from a drug are displayed as specified by the color legend. The raw data that is summarized in this Figure can be found in Supplementary Items 2–5 and Supplementary Table S2.

To investigate contributions to the cumulative neuropsychiatric signal beyond sedation and paradoxical responses in more detail, we analyzed the four psychiatric HLGs with the highest summed cumulative IC025 (Figure 3A) individually as shown in Figure 7. These comprise “psychiatric disorders nec,” “sleep disorders and disturbances,” “anxiety disorders and symptoms,” and “suicidal and self-injurious behavior.” Since sleep related disturbances occur both in the psychiatric and nervous system disorder SOCs, we merged these prior to the analysis (see Figure 2; Supplementary Figure S4). The individual AEs that contribute to each of the groups are provided in Supplementary Figures S4–S7 and Supplementary Table S2.

The HLG”psychiatric disorders nec” displays a higher fraction of total reports for males compared to females, and relatively balanced values are seen for the remaining three HLGs (Figure 7A). Diazepam has the highest cumulative signals for both sexes in the groups”psychiatric disorders nec” and “suicidal and self-injurious behavior.” Additionally, three of the five highest ranked drugs for both sexes are diazepam, alprazolam and clonazepam (Supplementary Table S2). Figure 7B provides for each drug the fractions of reports within each of the HLGs from panel A from the respective per drug 100% values. An interesting contributor to the male dataset “psychiatric disorders nec” is nordazepam, for which >13% of all associated AEs are from these four HLGs, Figure 7B. The group of “psychiatric disorders not elsewhere classifiable” comprises AE associations chiefly from abuse and withdrawal signs, see Supplementary Figure S5. We noted that nordazepam generally has a large signal, i.e., strong associations with “drug abuse,” “substance abuse” and related AEs. To look into these AEs more closely, we extracted the IC025 for each abuse-, addiction-and withdrawal relevant term from the neuropsychiatric SOCs on a per drug basis, and observed considerable heterogeneity there as well, see Supplementary Figure S8. In addition to nordazepam, oxazepam and lormetazepam have rather high IC025 values for most AEs related to abuse/addiction compared to loprazolam and flunitrazepam (triazulenone), which have only weak signals for males and no association for females at all. Notably, alprazolam is the only drug that is associated with all investigated AEs in that bucket (Supplementary Figure S8).

Clonazepam not only features a strong cIC025 in the “psychiatric disorders nec,” but is also among the top 5 ranked compounds in the HLGs “sleep disorders and disturbances,” “suicidal and self-injurious behaviors nec” and “anxiety disorders and symptoms.” In fact, for the male dataset, these four AE groups add up to nearly 10 % of all AEs this drug is associated with, Figure 7B.

In the merged HLG “sleep disorders and disturbances,” the strongest signals are seen for eszopiclone and zolpidem. For the case of eszopiclone, the fraction of reports falling into this HLG is also exceptionally high, as seen in Figure 7B. At the level of the individual adverse events that add up to the drugs’ cumulative signal in this group, eszopiclone is chiefly associated with signs of insomnia, while zolpidem is chiefly associated with various disturbances of sleep such as somnambulism and sleep related eating issues, see Supplementary Item 5. Interestingly, in sleep order and disturbances, two (male) and three (female) of the five highest ranked drugs are Z-drugs (Zolpidem, Eszopiclone, Zaleplon) as further described in Supplementary Table S2.

For the HLG “anxiety disorders and symptoms,” clonazepam is the top ranked drug for both sexes. For some drugs, striking differences in the normalized report numbers between sexes are seen (Supplementary Figure S7). Specifically, eszopiclone has a high cumulative signal of this HLG for females, but not for males. Vice versa, and, e.g., midazolam, tetrazepam and zaleplon feature stronger associations for males. The top AE observed in this HLG, namely agitation, (Supplementary Figure S7) is also contained in the neurological disorders nec. Group and is not specific for anxiety related issues. For individual drug-AE combinations, agoraphobia and panic signs add up to considerable signal strength for the case of clonazepam, in both sexes.

The selected examples highlight the fact that individual drugs have different association strengths with adverse events that belong to different groups of symptoms, and in part also between sexes. While the findings need to be interpreted with due care, the overall picture that emerges strongly suggests an unexpected degree of compound heterogeneity.

### 2.5. Sex differences

**Figure 8:**
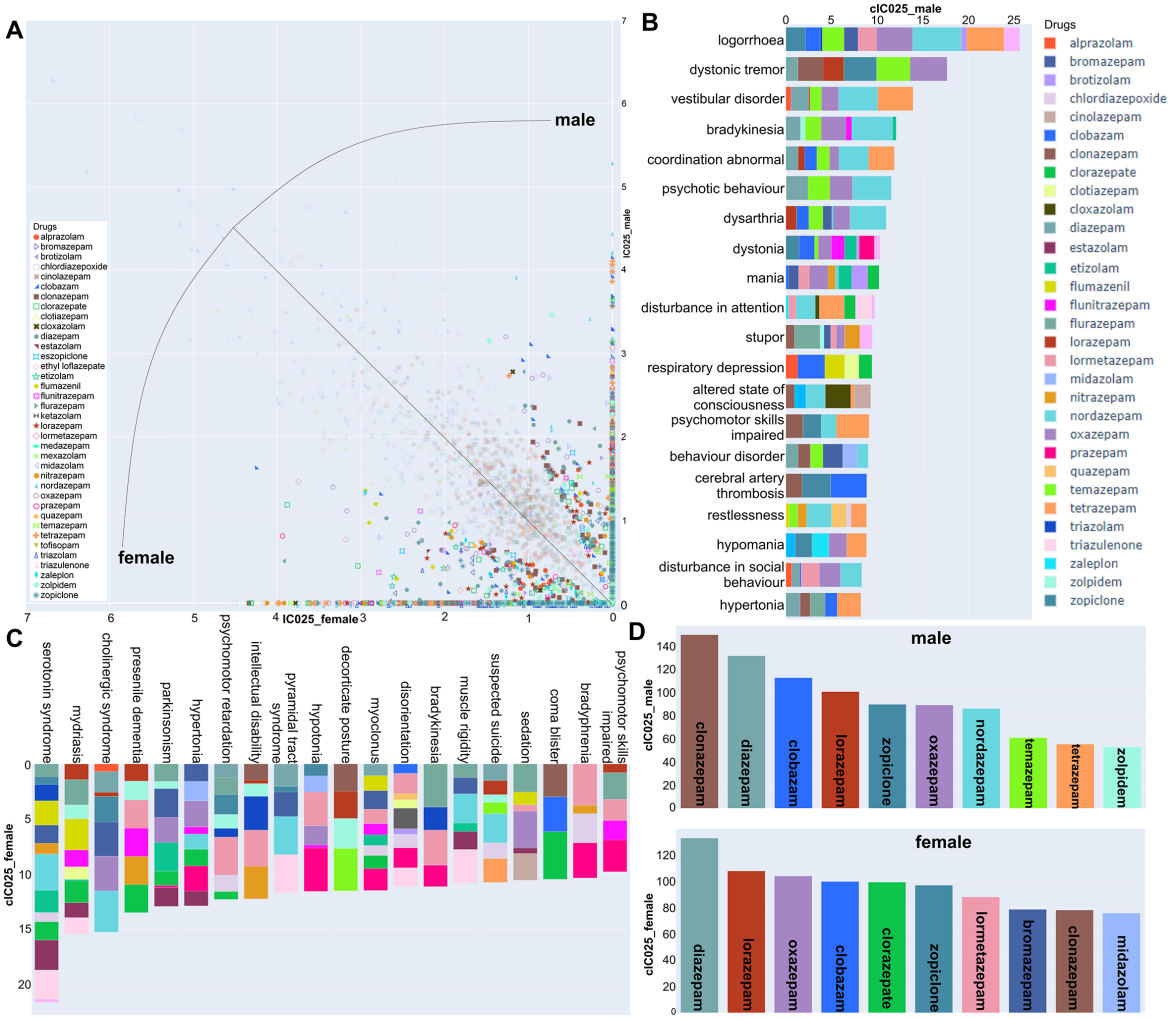
Sex differences in signal strength for neuropsychiatric AEs: **(A)** Scatter plot with all AE/drug pairs that exceed an IC025 ratio of 2:1 in either sex displayed in strong colors, pale colors for those <2. Points on the diagonal have equal IC025 for both sexes. **(B,C)** Top 20 neuropsychiatric AEs from the data of panel A above the 2:1 threshold toward one sex, are displayed (male: **B**; female: **C**) with the per drug contributions color coded. **(D)** The top 10 contributing drugs from the male and female data above threshold.

Given the occurrence of different signals for the two sexes in multiple datasets throughout our analysis, we next analyzed the data specifically with regard to sex differences in the neuropsychiatric SOCs on a per drug basis. In a first step, the merged neuropsychiatric data was visualized on a scatter plot where all drug/ AE pairs were plotted according to the respective sex-specific IC025 values, see Figure 8A. To capture those drug/ AE pairs, which have a stronger association in one sex, we filtered the data with an IC025 threshold of 2: 1 as displayed in Figure 8A. The further the ratio is from 1:1, the greater the distance between the data point and the diagonal. Thus, neuropsychiatric drug/AE events that occur in one sex only with a positive IC025, are reflected by points on the axes in Figure 8A. From the pools that have a signal ratio > 2:1 (and a signal in both sexes), we investigated the AEs with the highest cumulative IC025 from all the drugs per sex. The resulting top 20 for each sex are displayed in Figures 8B,C. The drug-AE pairs that have a signal only in one sex were also further analyzed by filtering for 20 drug-AE pairs with the highest IC025, see Supplementary Figures S9, S10. In addition, we identified the 10 drugs, which have the biggest contribution to adverse events in the same dataset, thus for all drug/AE pairs with a cIC025 ratio > 2:1 (Figure 8D).

Multiple psychiatric AEs are found in the data pool with stronger signals in one sex. Signs of paradoxical responses such as logorrhoea, mania, restlessness, and hypomania are seen for several drugs with a stronger association in males, but are absent from the top ranked AEs in the female >2:1 dataset. Nordazepam has an outstandingly strong signal for males not only for paradoxical responses, but a wide range of neuropsychiatric signs (see Supplementary Figure S11). Logorrhoea is associated with male reports exclusively for nordazepam, tetrazepam, and oxazepam see Supplementary Figure S9. For the data with a female: male ratio 2:1 or larger, the top ranked AEs are a mix of partly unspecific signs of neuropsychiatric changes. Among those AE-drug associations that are seen in females only, we note that anterograde amnesia is particularly strongly associated for clorazepate and lormetazepam, in the striking absence of an association for males (Supplementary Figure S10). Intrigued by this finding, and due to the relevance for illicit uses such as date rape of amnestic drugs, we mined the dataset for amnestic effects and confirmed the strikingly strong association for these two compounds, see Supplementary Figure S12. A minor point of interest here is that zolpidem is associated with a panel of amnestic AEs for both sexes, to a higher extent than the classical benzodiazepines.

Zolpidem is one of a few compounds with a stronger signal in the male:female >2:1 dataset, while the converse is true, e.g., for bromazepam and the already mentioned lormetazepam. Of the compounds which display stronger associations for males, the majority has more female reports in pool 2, thus, the difference in signal strength is likely a specific phenomenon. From the data that can be mined in this way from FAERS, findings concerning such sex-specific AE profiles of some drugs would be an interesting substrate for further pharmacoepidemiological and clinical follow up studies.

### 2.6. Physico-chemical descriptors

In total, pharmacovigilance data strongly suggests that the profiles of Bz-site ligands differ considerably in terms of the human *in vivo* effects that they can elicit, and that safety profiles for some of the drugs might be somewhat incomplete. The question of molecular drivers of such heterogeneity are manyfold, and deserve brief consideration to further align the pharmacovigilance derived effects with testable hypotheses for future research.

To get insight into molecular patterns of similarity and heterogeneity at the drug level, we evaluated all compounds based on physico-chemical and 3D properties. This was done as the integration of pharmacophore models and fingerprints in pharmacovigilance data analysis can reveal previously unknown safety concerns associated with a drug scaffold, and thus enable the implementation of measures to enhance drug safety. One such measure is to avoid certain moieties in drug development or to abstain from certain drugs to circumvent the occurrence of specific adverse events. Furthermore, this could allow for the exclusion of these derivatives in specific patient cohorts. A systematic view onto drug similarities and differences can also reveal unexpected cliffs in the structure–activity landscape and thus inform bed-to bench considerations for further drug development.

**Figure 9:**
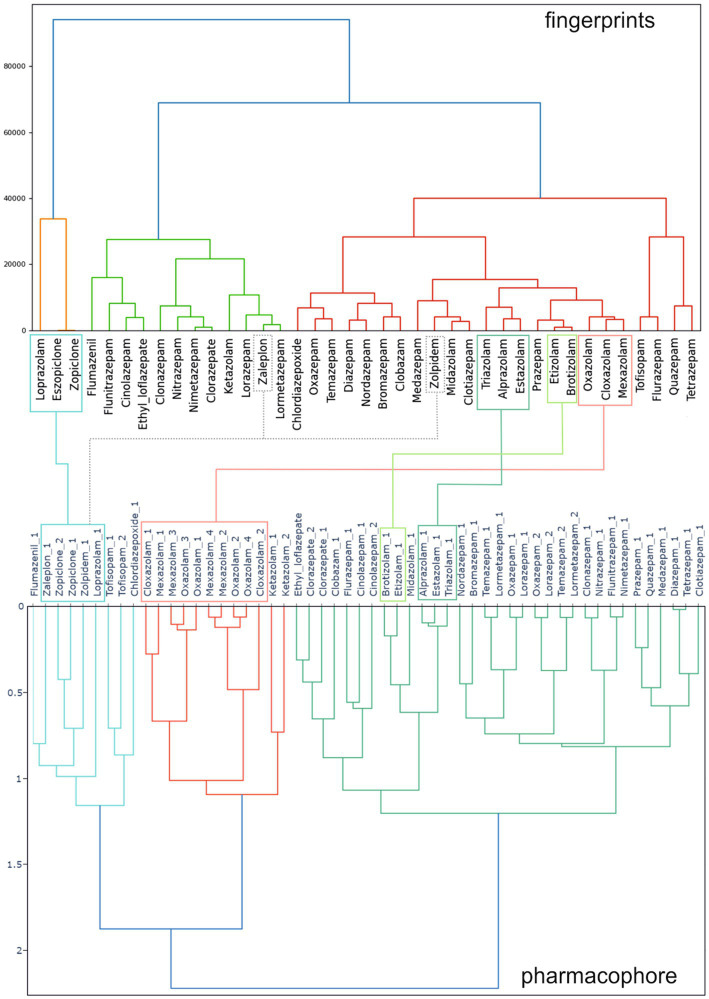
Clustering of 39 benzodiazepines and Z-drugs (from data pool 2 in Figure 2) based on their physico-chemical parameters and 3D properties. Upper panel: results of a ligand fingerprint analysis, which groups compounds based on their physico-chemical descriptors. Lower panel: results of a pharmacophore approach, which considers the size/shape and functional groups of compounds, thus is based chiefly on 3D properties of the molecules. Multiple stereoisomers of substances were considered for 3D- structure analysis and denoted by “_1” or “_2”: For many compounds, only one structure exists, for enantiomers with a single chiral center two molecules exist as is the case for zopiclone, and for mexazolam, four molecules exist. The original values were obtained from vROCS^®^ (for stereoisomer generation and shape/color calculations) and can be accessed in Supplementary Item 6. Connecting lines indicate some representative compounds that are grouped together by both, the ligand fingerprint analysis, and the pharmacophore approach. Dashed connecting lines indicate selected differences in clustering.

In the past, it has been difficult to establish structure–activity relationships for molecules targeting the GABAA receptors due to small changes in chemical scaffolds causing in part unexpected observed heterogeneity of structure–activity landscapes derived in heterologous expression systems, or even in preclinical and clinical *in vivo* outcomes (113, 114). The goal here was to approach the question of heterogeneity from the perspective of pharmacovigilance, and to relate the outcomes with ligand-based approaches. To accomplish this, we used two methods to describe the physico-chemical properties and 3D features of the molecules being studied. Firstly, we produced ligand fingerprints, which represent each molecule as a combination of recognized physico-chemical parameters, see Methods section. We then clustered the molecules, as shown in the upper panel of Figure 9. Secondly, we employed a pharmacophore model that incorporates both pharmacophore features (termed “color” by the used software) and molecular shape to group the substances based on their 3D orientation/size and functional groups, which are all critical factors in drug-protein target interactions. Their overlap in properties was used to group compounds, as shown Figure 9.

Our analysis revealed a more complex molecular landscape in the ligand fingerprint analysis than anticipated based on the 2D/3D-structure similarity of the compounds, which is consistent with the more traditional pharmacophore analysis. The pharmacophore demonstrated that all isomers of the “xazolam” compounds (cloxazolam, ketazolam, mexazolam, oxazolam) formed a distinct cluster due to their shared structural features. Similarly, the triazolo-compounds as well as the traditional 1,4-benzodiazepines such as diazepam and its metabolites also cluster together (Figure 9, lower panel). While ligand fingerprint analyses generally agree with the former, we resolved some unexpected clusters of compounds, such as zaleplon grouping together with lormetazepam (Figure 9, upper panel), which are structurally dissimilar based on 3D properties alone. Similarly, zolpidem formed a cluster closest to midazolam and clotiazepam, despite its distinct chemical 3D-body compared to the others. Thus, the ligand fingerprint analysis, based on various physicochemical properties, revealed a more nuanced picture, which is less intuitive but complements the 3D properties obtained by the pharmacophore results.

### 2.7. Complexity of on target effects

**Figure 10:**
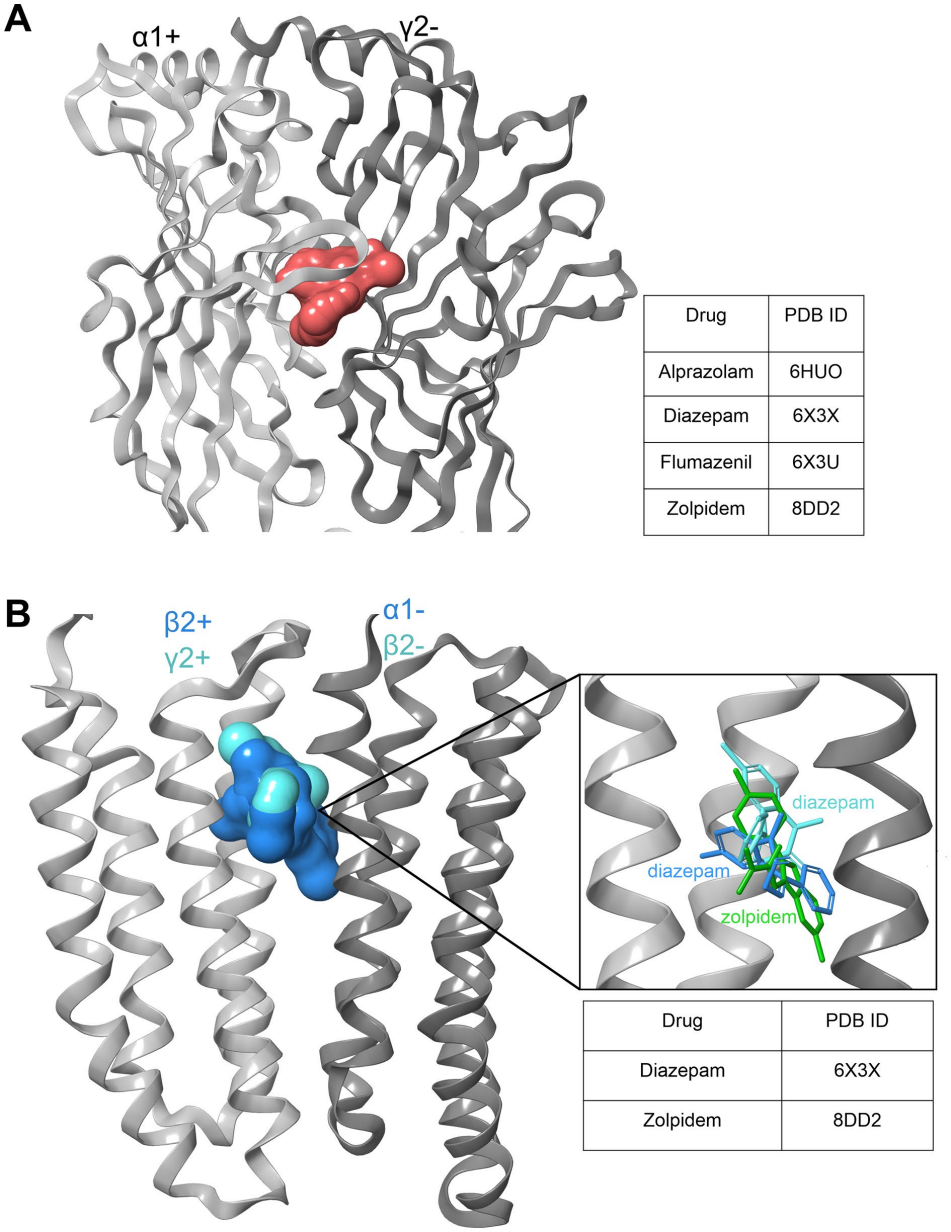
Benzodiazepine and zolpidem binding sites. **(A)** Extracellular high affinity binding site (BS) shared by BZDs and Z-drugs, which requires an α1,2,3 or 5 subunit as a principal component (+) of the BS and a γ1-3 as a complementary part (−) of the interface. Light gray: α1+; dark gray: γ2-; red surface: ligands bound to the binding site in various structures as listed in the table. **(B)** Diazepam and zolpidem low affinity binding sites within the transmembrane domain (TMD) of the GABAA receptor. Diazepam density has been resolved in both the α2+/β2-, and the β2+/α1- pockets within the interface at the upper TMD between the two subunits. Zolpidem was found only in the latter, in 8DD2 (117). Light gray: β2+/γ2+ subunit, dark gray: α1−/β2- subunit; ligand surfaces are superposed in blue and cyan. The insert shows the three ligands in superposition, color coded to emphasize the different binding modes that diazepam displays in the two sites, respectively.

The from FAERS signals suggested considerable compound heterogeneity is not too surprising in the light of the observed compound promiscuity at single GABAARs in the past combined with the existence of multiple homologous GABAAR subtypes (17, 115, 116). Recently structural data is accumulating that confirms and extends the existence of non-canonical binding sites and differential usage of binding modes, and thus provides structural correlates and hypotheses for effects specific to certain compounds. The current status of structural evidence is summarized in Figure 10.

The current structural evidence thus demonstrates several important points for the understanding of drug structure–activity relationships: (i) Compounds with a common chemical core can have different binding modes at the high affinity site, as demonstrated by the flumazenil binding mode that differs from the one observed for diazepam and alprazolam (118–120). (ii) The non-canonical sites that have been postulated on the basis of mutational studies are largely confirmed, and extended by the structural evidence (121, 122). The structural evidence again demonstrates distinct binding modes in these sites as well, as shown in Figure 9B (120). (iii) Biochemical evidence for further non-canonical binding sites, such as those observed at ECD β2+/γ2-interfaces (123) are supported by the observation of receptors that lack alpha subunits (17).

### 2.8. The complex relationship between chemical similarity and pharmacological trends

**Figure 11:**
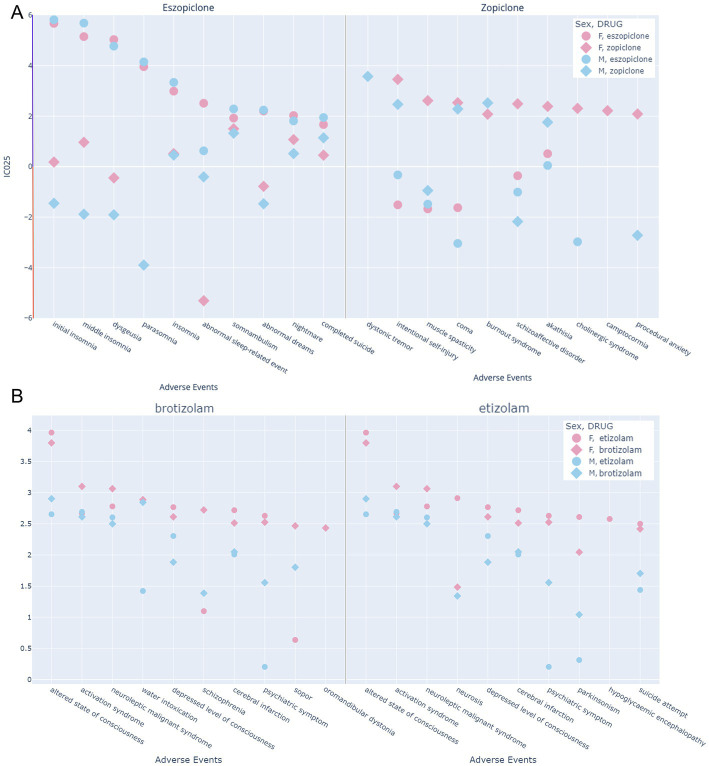
Top 10 neuropsychiatric AEs of four drugs to examine putative patterns of similarity induced by chemical similarity. **(A)** Eszopiclone and zopiclone: The left-hand side displays the top 10 adverse events of eszopiclone from the neuropsychiatric SOCs, as reflected by the IC025, and the corresponding values for zopiclone are displayed in addition. The right-hand side displays the top 10 adverse events of zopiclone from the neuropsychiatric SOCs, as reflected by the IC025, and the corresponding values for eszopiclone are displayed in addition. The y-axis reflects the IC025 value, which must be positive for an association between a drug and a reported adverse event. Data with negative values is taken from the data pool 1 prior to the disproportionality filter. **(B)** The left-hand side displays the top 10 adverse events of brotizolam from the neuropsychiatric SOCs, as reflected by the IC025, and the corresponding values for etizolam are displayed in addition. The right-hand side displays the top 10 adverse events of etizolam from the neuropsychiatric SOCs, as reflected by the IC025, and the corresponding values for brotizolam are displayed in addition. The y-axis reflects the IC025 value, which must be positive for an association between a drug and a reported adverse event. Data with negative values is taken from the data pool 1 prior to the disproportionality filter.

It is known that drug-protein interactions for ligands with chemical similarity form structure activity landscapes with “smooth” and “rugged” features, reflecting the interactions with binding sites that enable binding of similar molecules, and possess spatial features that lead to drops in activity due to small chemical changes. Thus, compounds that form clusters in chemical space are more likely to have overlapping pharmacological profiles – up to a degree. Intrigued by the seemingly dissimilar patterns of AEs found for zopiclone and eszopiclone, we analyzed these two compounds more closely along with another pair of chemically similar drugs, namely brotizolam and etizolam as displayed in Figure 11. For eszopiclone, the IC025 and fractional report share in the HLG “sleep disorders and disturbances” is outstandingly high. In contrast, zopiclone has no particularly strong association with any disturbance in sleep. This is intriguing as zopiclone is the racemic mix of eszopiclone and the presumed less affine/active R-enantiomer (124, 125).

First, it is very interesting to note that the top 10 AEs for eszopiclone and zopiclone display no overlap at all. As expected from the data presented in Figure 5, the majority of strong AE associations for eszopiclone are disturbances in sleep and sleep-related phenomena. They affect both sexes to a comparable degree. For these sleep related AEs, zopiclone in stark contrast has a mixed pattern of positive and negative associations. The top 10 AEs for zopiclone cover a broad spectrum of neuropsychiatric phenomena, some of which lack corresponding reports for eszopiclone altogether. For dystonic tremor, only male reports exist, and for muscle spasticity, a negative association for males and a robust positive signal for females are seen. In the case of intentional self-injury, which bears a strong association for zopiclone, the negative IC025 for eszopiclone strongly implies that this adverse event is associated specifically with zopiclone. These data, taken together, suggest that R-zopiclone is not simply a molecule with lower affinity (124), but rather can exert highly specific and dominant side-effects and can potentially overcome the paradoxical responses to eszopiclone on the sleep-related side effects. We also compared another pair of drugs which cluster together very closely in both fingerprints and pharmacophore features, namely etizolam and brotizolam. For this case, the FAERS profiles are highly similar as would be anticipated.

## 3. Discussion

As FAERS data has serious limitations that cannot be readily compensated for, pharmacoepidemiological studies would be needed to further substantiate or falsify the associations we identified. To address the question of robustness, we examined several selected drug-adverse event combinations that yield strong signals in our analysis with a semi-systematic search in the literature and in databases that rely in part on other means of evidence, such as the SIDER database (126) which chiefly utilizes product information, which in turn is derived from results obtained in appropriate clinical trials. The results of this analysis are provided in Table 1.

**Table 1:** FAERS signals and other evidence for selected drug-AE event pairs: from the SIDER database, side effects reported as “frequent” or “common” are indicated with +, others as (+), and effects not mentioned there are indicated with -, n/a stands for absent drugs.

AE-Drug	IC025	PRR	SIDER	Studies* in agreement
Seizures- clobazam	M: 4.54\F: 4.71	M: 25.64\F: 28.77	–	(127, 128)
Aggression-Nordazepam	M: 3.81\F: 2.00	M: 18.89\F: 9.78	n/a	
Alanine-Aminotransferase level abnormal – Nitrazepam	M: 4.93\F: 4.66	M: 82.06\F: 65.95	–	(129)
Propofol Infusion Syndrome-Midazolam	M: 4.84\F: 5.26	M: 58.51\F: 104.06	–	(130, 131)
Dysgeusia – Eszopiclone	M: 4.77\F:5.03	M: 30.5\F: 35.27	Undefined frequency	(132)

*Studies include clinical trials and papers reporting or analyzing clinical studies.

This compilation of converging pieces of evidence is far from comprehensive, but serves to demonstrate that strong associations derived from disproportionality analysis of large datasets often are confirmed in systematic studies.

With this study, we challenged the notion that benzodiazepines and Z-drugs are often considered to comprise a class of interchangeable drugs, apart from well accepted differences in pharmacokinetic properties. This is in striking contrast to anecdotal evidence and early preclinical literature (133). In order to examine data from human observations, we performed a comprehensive analysis of the pharmacovigilance data of BZ-site ligand associated AEs mined from the FDA adverse event reporting system. We included reports collected between 2004Q1 and 2021Q3. Those data suggest a diverse portfolio of AEs per compound, in part vastly different between individual compounds. This is partly reflected in product information and in the scientific literature, but systematic data and individual safety profiles are scarce. Thus, pharmacovigilance data is a precious source of human observations and can play a pivotal role in the identification of individual compound profiles.

Here we focused chiefly on neuropsychiatric AEs, as the BZ-site ligands are mostly classified as psychotropic substances, apart from a few antiepileptics. In the data extracted for neuropsychiatric MedDRA terms, only 11 of the 39 investigated drugs are responsible for more than 58% of the total neuropsychiatric ScIC025 (see Supplementary Figure S13) – this suggests that the currently available drugs show different dispositions to induce adverse neuropsychiatric signs. Our data suggests that this is not chiefly due to factors such as prescription bias, because we find drugs with small numbers of records (e.g., nordazepam) as well as compounds with high report numbers (e.g., diazepam) in the group with strong neuropsychiatric AE signals, but also highly prescribed substances such as triazolam or eszopiclone with a relatively low cumulative neuropsychiatric signal, see Figure 5 and Supplementary Figures S1, S2.

Outside of the neuropsychiatric SOCs, it was interesting to note though that in the MedDRA higher level group “investigations” we also found a high cIC025 signal. Interestingly, in records from females, changes in electrocardiogram parameters and in blood pressure are particularly strong compared to males (see Supplementary Figures S3, S14, S15).

Not surprisingly, multiple signs of over-sedation and related effects dominate the total neuropsychiatric ScIC025, closely followed by a very strong cumulative signal for signs indicative of paradoxical responses as seen in the group “neurological disorders nec,” see Figure 6. The observation that only few of the compounds strongly associate with signs of paradoxical responses might suggest that they are specific to certain drugs, and thus, their incidence should be quantified per drug and not for the class as a whole. At least at the level of pharmacovigilance we find clear signs for a high degree of drug specificity, which is in good agreement with preclinical work. The most startling observation in this category is probably the very strong association of eszopiclone, classified as a hypnotic drug, with different forms of insomnia and other sleep disturbance signs which is nearly absent in the zopiclone data (Figures 7, 11).

In the psychiatric higher-level groups, apart from expected effects such as signs of confusional states and impaired psychomotor responses due to over-sedation, we observe strong signals also for anxiety symptoms, and for self-injurious behaviors. In this group, we note another difference between zopiclone and eszopiclone: Self-injurious behaviors are strongly associated with zopiclone only (see Figure 11). In total, the FAERS data suggests considerable drug heterogeneity. This applies not only to unwanted effects of medically used BZ-site ligands, but also issues related to non-medical drug use. We extracted the IC025 values per drug for signs and symptoms of dependence, drug abuse, and for withdrawal symptoms (Supplementary Figure S8). While this dataset needs to be interpreted with due care and may be biased by many confounding factors, it does feature considerable drug heterogeneity that is worthy of further investigation.

Non-medical drug use can be recreational due to desired drug effects, e.g., as downers or to enhance effects of other psychoactive substances, or for illicit purposes such as “date rape” drug administration. In this context, amnestic effects are of particular interest. We noted that anterograde amnesia is associated with female records exclusively for the case of lormetazepam and clorazepate with an IC025 > 3 (Supplementary Figures S10, S12). Only 17 (of 39 drugs in our pool of disproportionately strong associations) drugs were found to be associated with any amnestic effects, and for example among the Z-drugs, zaleplon has none. These findings suggest that the individual drugs show also considerable heterogeneity with respect to properties that are compatible with abuse as date rape drugs. While the limitations of pharmacovigilance data fully apply, further follow up of such hints toward drug heterogeneity should stimulate systematic investigations.

In this study we specifically identified pronounced drug differences in AE event profiles of a substantial number of compounds. Taking into consideration that benzodiazepines are prescribed approximately twice as often to women as to men in the US, a similar ratio of adverse event reports in the FAERS database would be anticipated in absence of sex specific factors involved. However, additional layers of complexity have to be taken into consideration that limit data interpretation: (1) the possibility that adverse reports are more often reported for a specific sex, even if they occur in the other sex as well (2) that some benzodiazepines are prescribed more often than others for women (such as for anxiety disorders) and might bias the reports therefore for certain indications toward one sex, (3) the dark figure of individuals abusing benzodiazepines without prescription, which is reported to be higher in men, (4) the missing total number of prescriptions for a specific drug and sex in the FAERS database from which the reports result, and (5) the reports resulting from prescription for different indications, and thus different dosages which information is mostly lacking (6) the reports based on illicit use that is lacking prescription and is combined with other substances such as opioids and alcohol often and (7) others.

However, preclinical and *in vitro* research provides some hints though why some compounds may display sex differences that are not due to data bias: Supra- additive effects with endogenous cannabinoids (37) and (neuro-) steroids (134) have been observed for benzodiazepines *in vitro*. Recent studies propose native GABAA receptors to possibly harbor endogenous allopregnanolone before the addition of benzodiazepines (38).

We attempted to correlate molecular properties with FAERS derived drug profiles which we identified. Overall, we found very little evidence for any correlations between the chemical compound properties and their pharmacovigilance fingerprints. The limitations of a ligand-based approach to structure–activity relationships is impressively demonstrated by the vastly different FAERS profiles of eszopiclone and zopiclone. Our data implies the coexistence of two phenomena: As evidenced by the highly overlapping chemical and FAERS profiles of etizolam and brotizolam (see Figure 11), highly similar compounds can share most key properties as drugs – and in contrast, steep cliffs in structure activity landscapes can occur as well as appears to be the case for S- and R- zopiclone. The latter phenomenon has its structural correlation in the multitude of binding sites with which each molecule can interact with individual affinity and efficacy. It has long been hypothesized that a multitude of distinct receptor subtypes with unique binding sites are and mediate the broad range of *in vivo* effects that are observed for benzodiazepine site ligands as reviewed in (135). In line with this, research from rodent models on subtype specific pharmacology has had limited translation success (113), which is at least in part owed to different transcriptomes of neuronal cell types, e.g., in the limbic system and discrepancies in regio-specific subunit expression between animals and humans (136). Hence, there is accumulating evidence that not only the so-called “high affinity” binding sites of these drugs contribute to pharmacologically relevant effects, but that additional binding sites that are shared in part with general anesthetics also contribute to the observed *in vivo* spectrum of effects. Differences in compounds’ ability to utilize these interaction sites will lead to specific pharmacodynamic profiles. It is already clear that the tendency for individual BZ-site ligands to occupy additional sites apart from the canonical high affinity sites is different among compounds (120, 122), which can be expected to impact massively on the spectrum of *in vivo* effects due to the near complete lack of isoform- differences in the low affinity sites (137). A recent surge in structural findings allows an updated view of known and putative allosteric sites by which wanted and unwanted pharmacological effects are potentially mediated, as summarized in Figure 10.

Moreover, even the heterogeneity of compound binding and effects at the canonical sites is vastly understudied: The pharmacology for γ1 and γ3 is very incomplete, and the high expression level of the γ1 subunit in human limbic system structures might account for highly specific drug effects for substances that act on γ1- containing receptors (133). An added layer of complexity comes from the modulatory efficacy, which can range per compound, substance concentration and high affinity site from strong GABA enhancing (PAM) effects to strong GABA diminishing (NAM) effects (133, 138–140). For most approved benzodiazepines and Z-drugs, data of their modulatory effect in the major receptor subtypes is completely lacking and PAM effects are assumed, with the exception of the “antagonistic” chiefly silent modulator flumazenil. Beyond the vast diversity of allosteric sites used by “Bz-site-ligands” on GABAARs, off-target effects certainly may be drivers of individual drug effects as well, even though broad panel CNS- target assays indicate that most of these compounds have fewer off-targets compared to many other CNS therapeutics.

In summary, this study provides insights into the pharmacological properties of BZD compounds and Z-drugs and helps to inform clinical decision-making and drug development in this area. The analysis of the FAERS dataset and the application of ligand fingerprint and pharmacophore analyses reveal a more nuanced picture of the heterogeneity of BZ-site ligands, which can help to identify potential therapeutic uses and adverse effects as well as shape clinical studies on this topic in the future. The FAERS profiles of many compounds suggest sex-specific side effects.

Our analysis produced strong drug-AE associations. While pharmacovigilance data cannot confirm alerts nor offer mechanistic interpretations, we hope our findings stimulate follow up research, and potentially adaptations of prescription practice to meet modern standards of sex-specific care. If appropriate clinical studies can confirm some of the associations derived from the FAERS dataset, product information and subsequently also the legal classification of individual compounds could conceivably be adjusted to account for increased risks of unwanted effects by the addition of specific warnings to product information.

## 4. Materials and methods

### 4.1. Data mining

Four publicly available sources were used to generate a list of benzodiazepines and Z-drugs: Drugbank (141), Wikipedia (142, 143), and Wikidata (144). To accomplish this, different data extraction techniques were utilized for each source. To collect pharmaceuticals from Drugbank, for example, a Python script was used to filter compounds associated with each GABAAR subunit, and this list was then manually screened for benzodiazepines and Z-drugs. We used SPARQL to search Wikidata for drugs related to any of the 19 components. We also obtained benzodiazepines from two Wikipedia pages (142, 143). The final list included 173 benzodiazepines and Z-drugs.

### 4.2. FAERS analysis

To conduct the pharmacovigilance analysis of benzodiazepines and Z-drugs, a FAERS (FDA Adverse Event Reporting System) dataset was utilized, which was taken from Khaleel et al. (105). This dataset covers adverse event reports from Q1 2004 to Q3 2021. Initially, the dataset was divided into male and female subsets. Records with unknown sex were removed. Records from female reports that deal with occurrences of the offspring were removed by manual curation if the need arose. Afterwards, a disproportionality analysis was performed for each drug-adverse event pair in both datasets.

Disproportionality analysis was used to assess the association strength between drug use and reported unfavorable outcome (or adverse event, AE) (100–102). For each drug-adverse event pair, the information component (IC), 95% confidence interval of IC (IC025) (100, 102), proportional reporting ratio (PRR), and reporting odds ratio (ROR) was calculated (101, 105). The IC was employed to evaluate the likelihood of genuine values falling within an assigned range. The Uppsala Monitoring Centre developed and validated this approach using Bayesian neural networks to create the information component IC (111, 145), which represents the logarithmic base 2 of observed/expected ratios and is commonly used for analyzing WHO databases (100, 102, 146). In numerous studies conducted by both UMC and other researcher groups, IC025 has been utilized as a benchmark for identifying positive drug-adverse event connections (102, 104, 147–151). Further investigations employ the PRR measure with the IC025 and necessitate a minimum of 5 observations to ensure an affirmative signal (107–110), which we followed in this work. All relevant records were extracted for the 173 drugs on our drug list belonging to the benzodiazepines and Z-drugs categories from the dataset and examined the system organ classes (SOCs) and higher level groups (HLGs) within the MedDRA. For this MedDra Version 22.1 was used and only adverse events have been considered which could be identified in this MedDra version. The level of individual AEs was analyzed where appropriate by application of filters. Cumulative IC025 values, as well as relative and absolute report numbers were obtained as appropriate sums from the filtered records.

**Table:** 

	Drug	All other drugs	Total
Adverse event	a	b	a + b
All other adverse events	c	d	c + d
Total	a + c	b + d	n = a + b + c + d

a = Reports of the drug of interest with the adverse event of interest. b = Reports of all other drugs with the adverse event of interest. c = Total drug reports of all other adverse events. d = Total reports of all other drugs with all other adverse events.


$$\text{Proportional Reporting Ratio}\;\left(PRR\right)=\frac{\frac{a}{\left(a+c\right)}}{\frac{b}{\left(b+d\right)}}$$

$$\text{Information component}\;\left(IC\right)=\log_{2}\frac{a+0.5}{a_{\exp}+0.5}$$

$$a_{\exp}=\frac{\left(a+b\right)*\left(a+c\right)}{\left(a+b+c+d\right)\;}$$

$$IC025=IC-3.3*{\left(a+0.5\right)}^{-\frac{1}{2}}-2*{\left(a+0.5\right)}^{-\frac{3}{2}}$$

$$\text{Reporting Odds Ratio}\;\left(ROR\right)=\frac{\frac{a}{c}}{\frac{b}{d}}$$

For all calculations, the python libraries pandas 2.8.2 and numpy 1.22.4 have been used with python 3.10. All the figures with the FAERS results have been generated with plotly 5.13 with python 3.10.

Calculation which has been used in Figure 4B:

*Calculation:*


$$\text{Drug percentage}=\frac{\text{cumulative}\;IC025\;\text{of}\;a\;\text{specific drug in}\;SOC}{\text{sum}\;\text{of cumulative}\;IC025\;\text{of}\;\text{all}\;\text{drugs in}\;SOC}*100.$$

Calculation which has been used in Figure 7B:

*Calculation:*


$$\text{Reports percentage}=\frac{\text{total reports of drug in}\;HLG}{\text{total reports of drug}}*100$$

### 4.3. Ligand based methods and creation of plots

#### 4.3.1. Fingerprints

To calculate multiple molecular fingerprints from the sdf files of 39 drugs, Python 3.10 and PyBioMed 1.0 library were utilized which included moe, ghosecrippenfingerprint, cats2d, connectivity and topology. Further analysis was carried out through principal component analysis (PCA) using scikit-learn 1.0.2 library while plotly version 5.13 was used to create the dendrogram for graphical representation of the results.

#### 4.3.2. 3D-structure similarity analysis

3D structures of the investigated drugs were retrieved from PubChem (152) as individual SD-files which were then merged to obtain a single file for further processing. The drug data set SD-file was then processed by the software Flipper (153) [version 3.1.1.2, executed with the following settings: (enumEZ true-enumNitrogen false-enumRS true-enumSpecifiedStereo true-warts true)], to enumerate all possible stereoisomers of drugs having one or more stereocenters. In the output file (SD format) the stereoisomers of the drugs are distinguished by a name suffix that consists of an underscore followed by the number of the stereoisomer. The stereoisomer enriched drug data set was then subjected to conformer ensemble generation using the software OMEGA (153, 154). Default settings were used for all parameters except for the energy-window (−ewindow) and the RMSD-threshold (−rms) setting for which values of 20.0 and 0.25, respectively, were chosen to obtain a more detailed representation of the conformational space of the compounds. The generated conformers were stored as a single SD-file which then served as input for molecular shape-based drug similarity calculations using the program ROCS (154). In order to calculate shape similarity values for all possible drug stereoisomer pairs, the obtained multi-conformer SD-file was specified both as an input file for the query (= reference) structures (−query) as well as for the evaluated database molecules (−dbase). For other ROCS parameters the preset defaults were used with the exception of the multi-conformer query flag, which was set to false (−mcquery false), the single-conformer database flag, that was set to true (−scdbase true) and the “per query structure generated ROCS reports” were merged into a single report output file (−report one). By means of a Python script (‘report_to_dist_matrix.py), the obtained ROCS report file was then converted to ‘shape distance’ matrices based on the listed ColorTanimoto, ShapeTanimoto and TanimotoCombo scores of all drug stereoisomer pairs. Only the TanimotoCombo was used for further processing. For a drug stereoisomer pair *ij*, the shape distance *D_ij_*, which can adopt values in the range [0, 1], is calculated from the maximum similarity score *S_ij_* that was encountered among all evaluated conformer pairings as follows:

D_ij_ = 1 – S_ij_/S_max_

*S_max_* denotes the maximum value the particular ROCS similarity score can reach (1.0 for Color- and ShapeTanimoto, 2.0 for TanimotoCombo) and is used to scale the similarity score *S_ij_* to the range [0, 1].

The combo scores of ROCS have been taken for further analysis. The dendograms have been created using python 3.10 with the library plotly 5.13.0.

### 4.4. Analysis of structural data

The PDB was mined for all structures of GABAA receptors with any of the analyzed BZ-site ligands in the complex. The resulting structures [8DD2 (subunit rendering), 6X3X (118), 6HUO (119), 6X3U (120)] were superposed and rendered with Schrödinger/Maestro Version 13.1.141.

## Data availability statement

The original contributions presented in the study are publicly available. This data can be found at: https://github.com/FilipKon/Drug_Diversity_FAERS.

## Author contributions

FK and TS performed experiments. FV, FK, ID, MW, MK, TS, and ME contributed to writing. FV, ME, MW, ID, and FK contributed to shape the scope of the manuscript and literature research. ID and MW were involved in the choice of drugs. FV and ME designed experiments. ME provided funding and supervised the work. All authors contributed to the article and approved the submitted version.

## Funding

The authors gratefully acknowledge financial support from the Austrian Science Fund in the MolTag doctoral programme FWF W1232 and from the European Community: The NeuroDeRisk project has received funding from the Innovative Medicines Initiative 2 Joint Undertaking under grant agreement No. 821528. This Joint Undertaking receives support from the European Union’s Horizon 2020 research and innovation programme and EFPIA.

## Conflict of interest

The authors declare that the research was conducted in the absence of any commercial or financial relationships that could be construed as a potential conflict of interest.

## Publisher’s note

All claims expressed in this article are solely those of the authors and do not necessarily represent those of their affiliated organizations, or those of the publisher, the editors and the reviewers. Any product that may be evaluated in this article, or claim that may be made by its manufacturer, is not guaranteed or endorsed by the publisher.

## Figures and Tables

**Table:** 

ATC	Anatomical Therapeutic Chemical Classification System
AE	Adverse Event
AUD	Alcohol Use Disorder
BS	Binding site
BZD	Benzodiazepine
cIC025	Cumulative IC025
CNS	Central Nervous System
CYP	Cytochrome P450 System
DAWN	Drug Abuse Warning Network
FAERS	FDA Adverse Event Reporting System
GABAAR	GABAA Receptor
HLG	Higher Level Group
IC	Information component
OC	Oral Contraceptives
PRR	Proportional Reporting Ratios
PT	Preferred Term
ROR	Reporting Odds Ratio
ScIC025	Summed cumulative IC025
SOC	System Organ Class
+	Principal component of the binding site
-	Complementary component of the binding site
