# Supplementary material for: Navigating the complex landscape of benzodiazepine- and Z-drug diversity: insights from comprehensive FDA adverse event reporting system analysis and beyond
Source: Front Psychiatry. 2023 Jun 23;14:1188101. doi: 10.3389/fpsyt.2023.1188101 (PMC10345211; doi:10.3389/fpsyt.2023.1188101)
Supplement: Supplementary file 6 [file Data_Sheet_1.PDF]

Supplementary Material

Navigating the complex landscape of benzodiazepine- and Z-drug diversity: Insights from comprehensive FAERS analysis and beyond

Filip Koniuszewski[1]† Florian D. Vogel[1]†, Irena Dajić[2], Thomas Seidel[3], Matthäus Willeit[2], Margot Ernst[1*]

*** Correspondence:**

Margot Ernst, Department of Pathobiology of the Nervous System, Center for Brain Research, Medical University Vienna, Spitalgasse 4, Vienna 1090, Austria.

1 Supplementary Data

Supplementary Item 1

Benzodiazepine_list.xlsx

List of all benzodiazepine names which have been taken into account for the filtering process.

Supplementary Item 2

FAERS_Total_reports.xlsx

The total reports for all benzodiazepine and z-drugs, which have been found in the FAERS data set before and after the filtering process.

Supplementary Item 3

FAERS_data_female.xlsx

The FAERS data, consisting of drug, adverse event, and report information exclusively for females, presented in an csv file which includes the MedDRA system organ classes, higher level group terms, and values for IC, IC025, PRR, and ROR.

Supplementary Item 4

FAERS_data_male.xlsx

The FAERS data, consisting of drug, adverse event, and report information exclusively for males, presented in an csv file which includes the MedDRA system organ classes, higher level group terms, and values for IC, IC025, PRR, and ROR.

Supplementary Item 5

Top4_psychiatricdisorders.xlsx

From Figure 7A the highest ranked drug with the AEs that contribute to the cIC02. The excel file is separated for each drug one sheet. Additional to the IC025 values the PRR and reports are added.

Supplementary Item 6

vrocs_results.csv

Supporting data for Figure 10: Raw data obtained from VROCS in the form of a csv. Excel file.

2 Supplementary Figures and Tables

2.1 Supplementary Figures

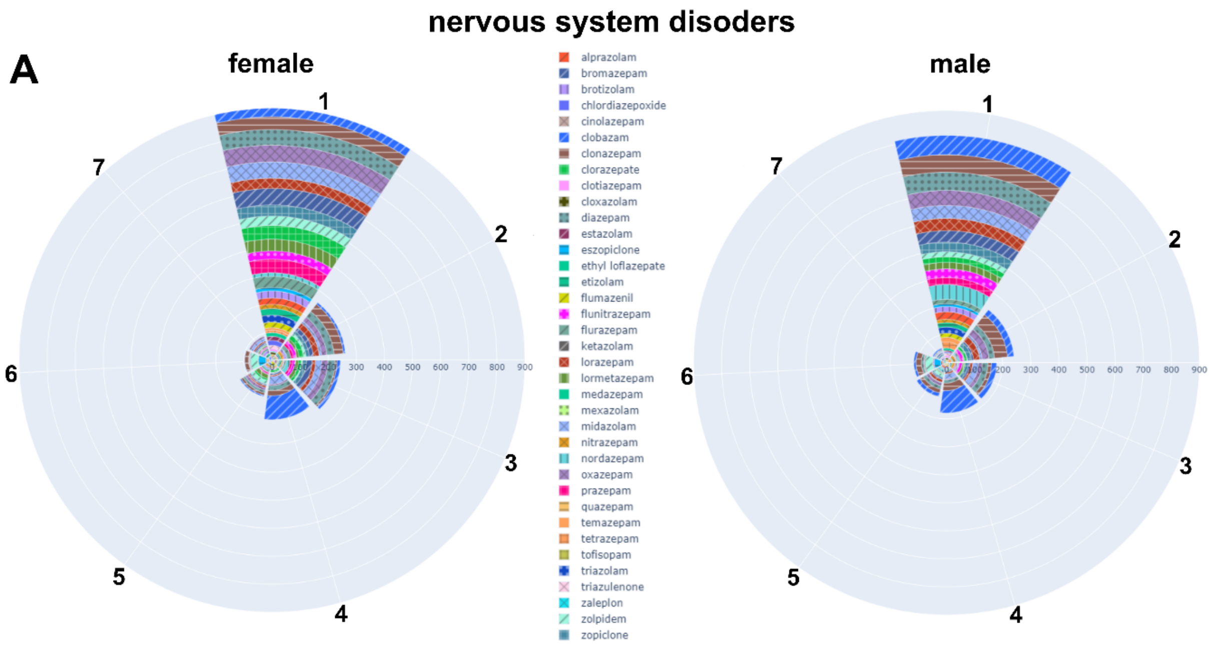

nr	HLG	nr	HLG	nr	HLG
1	neurological disorders nec	2	movement disorders (incl parkinsonism)	3	neuromuscular disorders
4	seizures (incl subtypes)	5	mental impairment disorders	6	sleep disturbances (incl subtypes)
7	central nervous system vascular disorders				

Supplementary Figure 1: *Overview of the SOC nervous system disorders. (A) Cumulative IC025 values for all HLGs and both sexes are shown in polar bar charts with the respective cumulative IC025. Each drugs' contribution is a patterned segment as indicated by the legend. (B) All HLGs including total report numbers for both sexes are depicted in bar charts. The second image is the enlarged version of the boxed part.*

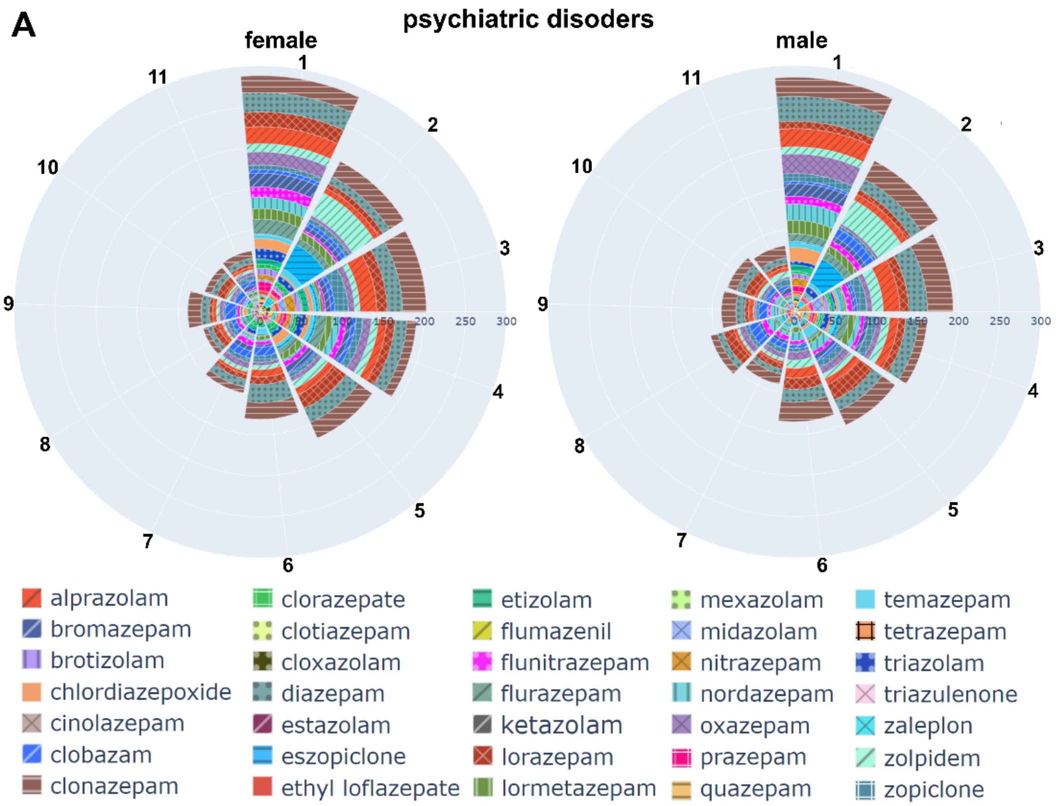

1	psychiatric disorders nec	2	sleep disorders and disturbances	3	anxiety disorders and symptoms
4	suicidal and self-injurous behaviours nec	5	disturbances in thinking and perception	6	personality disorders and disturbances in behaviour
7	psychiatric and behavioural symptoms nec	8	communication disorders and disturbances	9	mood disorders and disturbances nec
10	changes in physical activity	11	depressed mood disorders and disturbances		

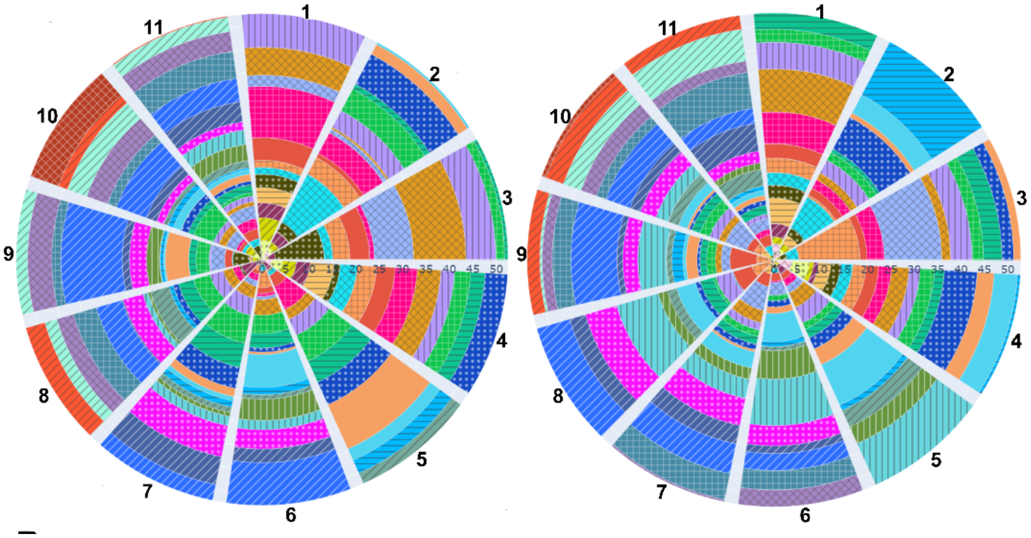

Supplementary Figure 2: *Overview of the SOC psychiatric disorders. (A) Cumulative IC025 values for all HLGs and both sexes are shown as polar bar charts with the respective cumulative IC025.*

Each drugs' contribution is a patterned segment as indicated by the legend, and the contributing HLGs are identified in the table. For all HLGs the total report numbers for both sexes are depicted as bar chart. The lower charts are enlarged view of panel A (range 0-50). The raw number of reports per sex for each drug from data pool 2 can be found in Supplementary Item 3 and 4.

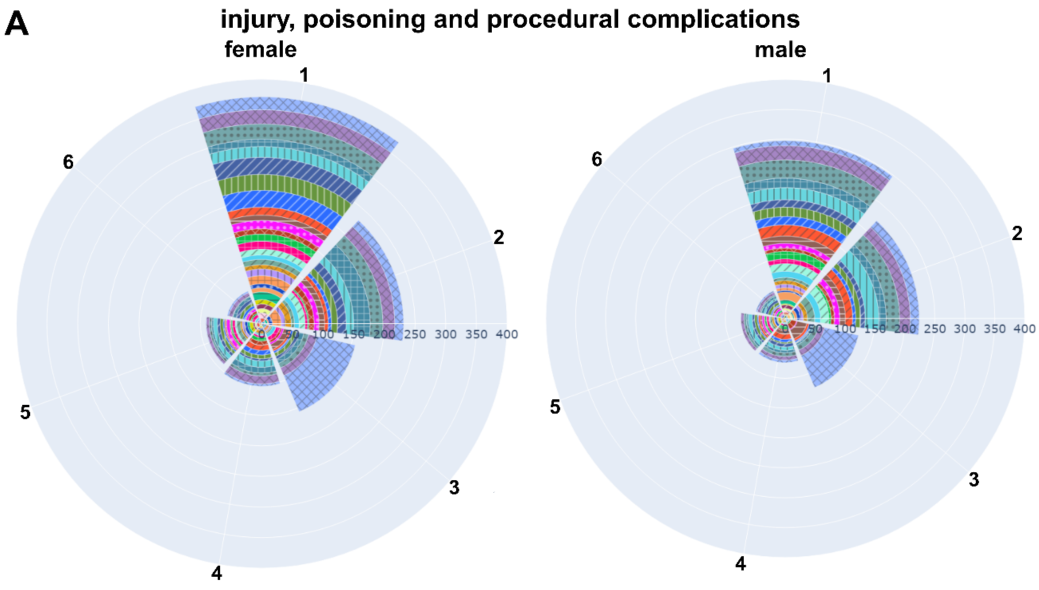

1	exposures; chemical injuries and poisoning	2	injuries nec	3	procedural related injuries and complications nec
4	medication errors and other product use errors and issues	5	overdoses and underdoses nec	6	injuries by physical agents

alprazolam, bromazepam, brotizolam, chlordiazepoxide, clobazam, clonazepam, clorazepate, clotiazepam, cloxazolam, diazepam, estazolam, eszopiclone, ethyl loflazepate, etizolam, flumazenil, flunitrazepam, flurazepam, ketazolam, lorazepam, lormetazepam, medazepam, midazolam, nitrazepam, nordazepam, oxazepam, prazepam, quazepam, temazepam, tetrazepam, tofisopam, triazolam, triazulenone, zaleplon, zolpidem, zopiclone

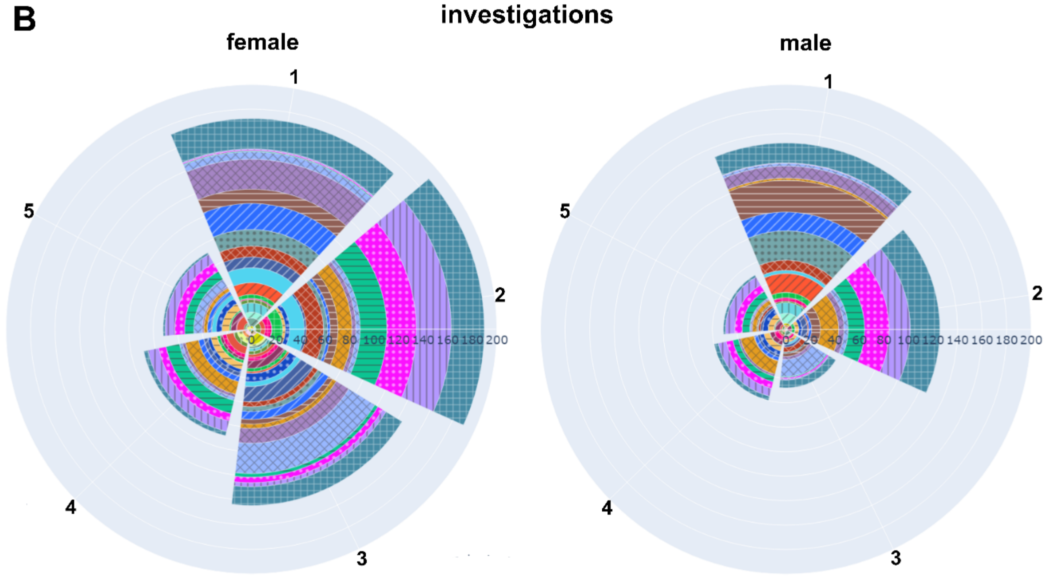

1	toxicology and therapeutic drug monitoring	2	haematology investigations (incl blood groups)	3	cardiac and vascular investigations (excl enzyme tests)
4	hepatobiliary investigations	5	enzyme investigations nec		

Supplementary Figure 3: *Overview of the SOC injury, poisoning, procedural complications and investigations. (A) Cumulative IC025 values for all HLGs and both sexes are shown in polar bar charts with the respective cumulative IC025 in the SOC injury, poisoning, procedural complications. Each drugs' contribution is a patterned segment as indicated by the legend. (B) Cumulative IC025 values for all HLGs and both sexes are shown in polar bar charts with the respective cumulative IC025. Each drugs' contribution is a patterned segment as indicated by the legend in the SOC investigations.*

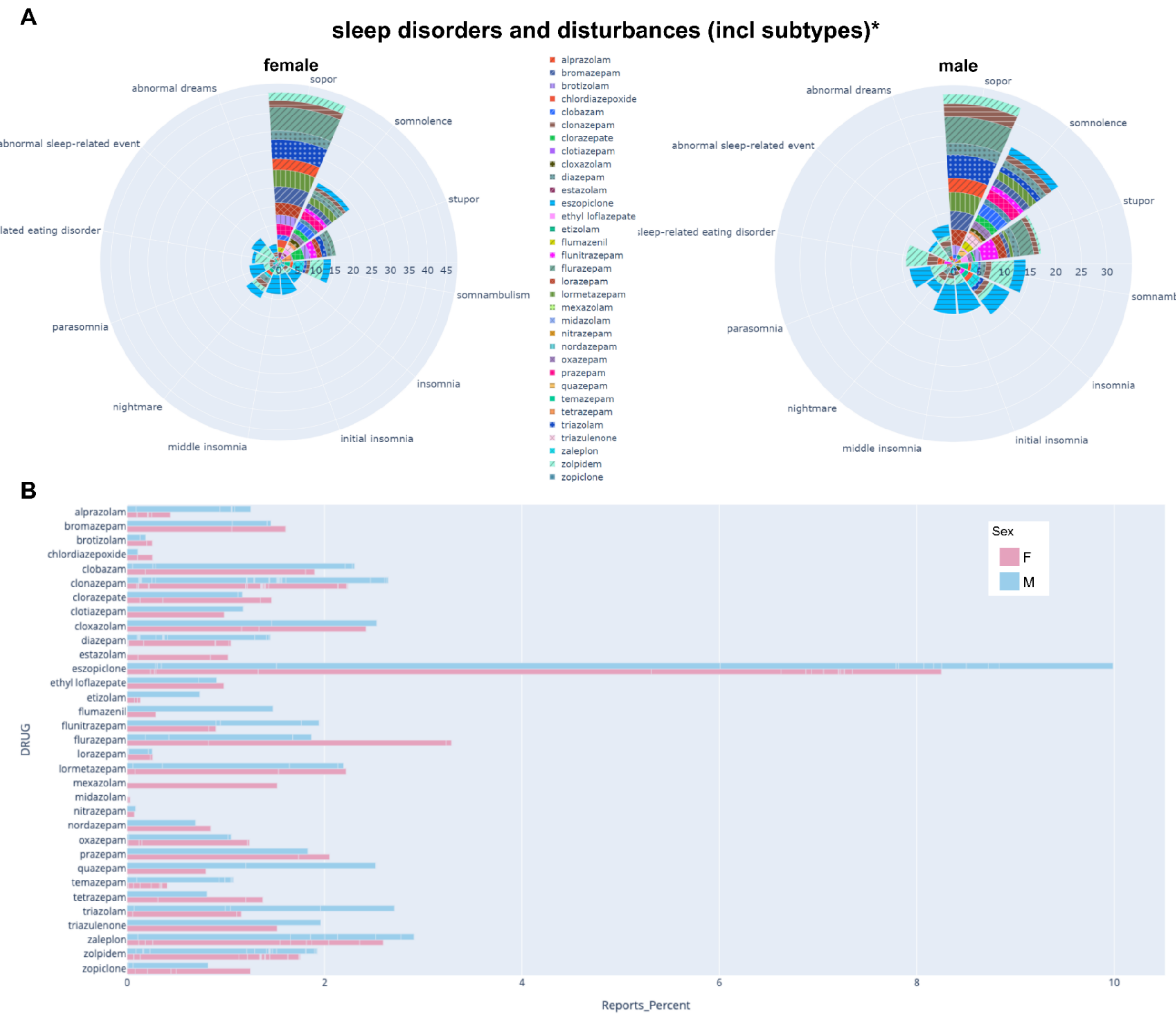

Supplementary Figure 4: *Overview of the merged HLG comprising sleep disorders and disturbances (incl subtypes). The group sleep disorders and disturbances is derived from the SOC "psychiatric disorders", and from the HLG sleep disturbances (incl subtypes) from the SOC "nervous system disorders". Duplicates have been deleted. (A) AEs with a cumulative IC025 for both sexes combined >30 are displayed for both sexes individually. IC025 values per drug are depicted in a polar bar plot. (B) All drugs with AE reports in that category are displayed for each sex as a fraction of the total reports for this drug.*

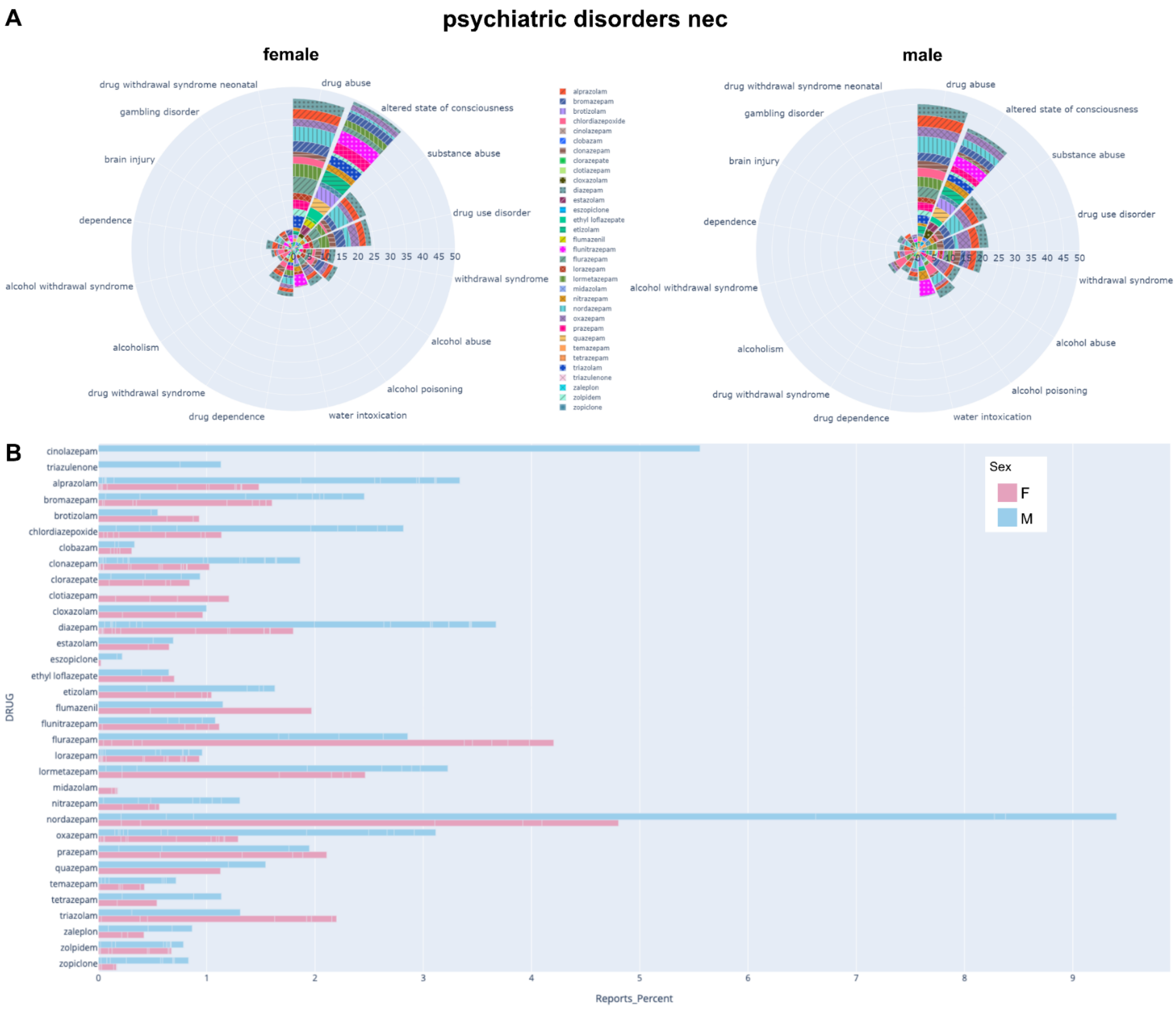

Supplementary Figure 5: *Overview of the HLG psychiatric disorders nec. (A) AEs with a cumulative IC025 for both sexes combined >30 are displayed for both sexes individually. IC025 values per drug are depicted in a polar bar plot. (B) All drugs with AE reports in that category are displayed for each sex as a fraction of the total reports for this drug.*

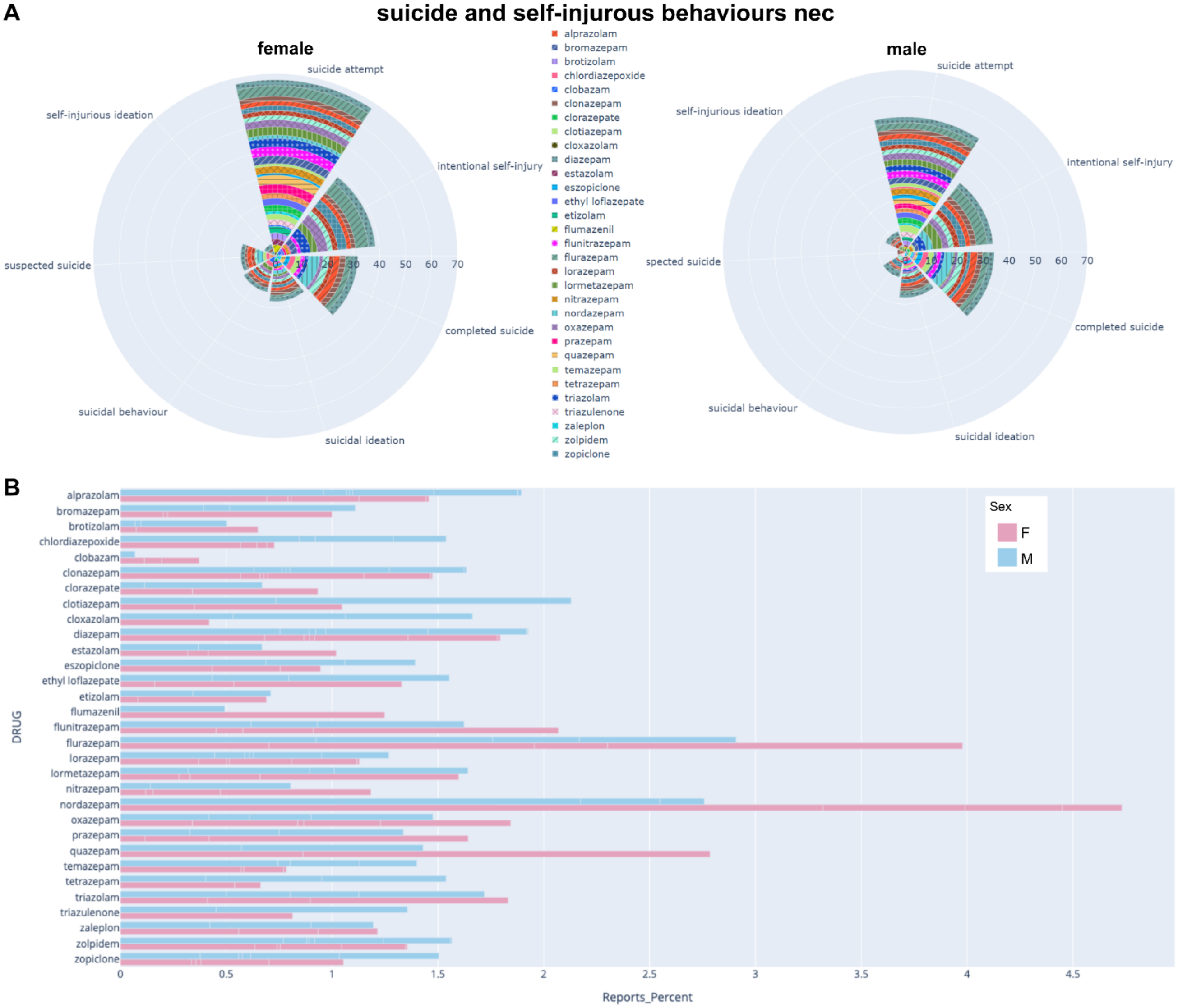

Supplementary Figure 6: *Overview of the HLG suicide and self-injurous behaviours nec. (A) AEs with a cumulative IC025 for both sexes combined >30 are displayed for both sexes individually. IC025 values per drug are shown in polar bar plots. (B) All drugs with AE reports in that category are displayed for each sex as a fraction of the total reports for this drug.*

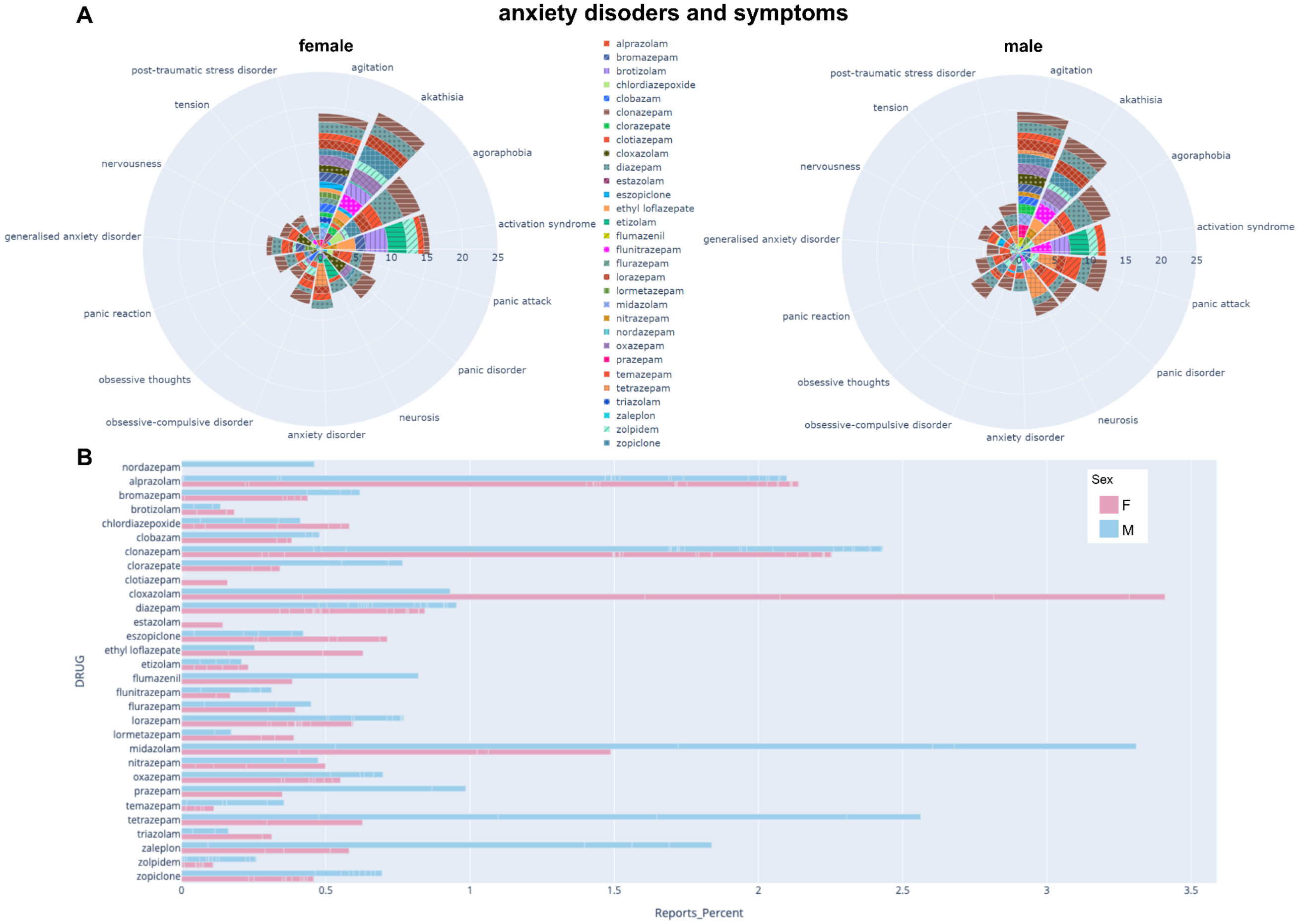

Supplementary Figure 7: *Overview of the HLG anxiety disorders and symptoms. (A) AEs with a cumulative IC025 for both sexes combined >30 are displayed for both sexes individually. IC025 values per drug are depicted in a polar bar plot. (B) All drugs with AE reports in that category are displayed for each sex as a fraction of the total reports for this drug. It is interesting to note the pronounced sex differences for several drugs, with both female and male bias in the normalized reports (panel B).*

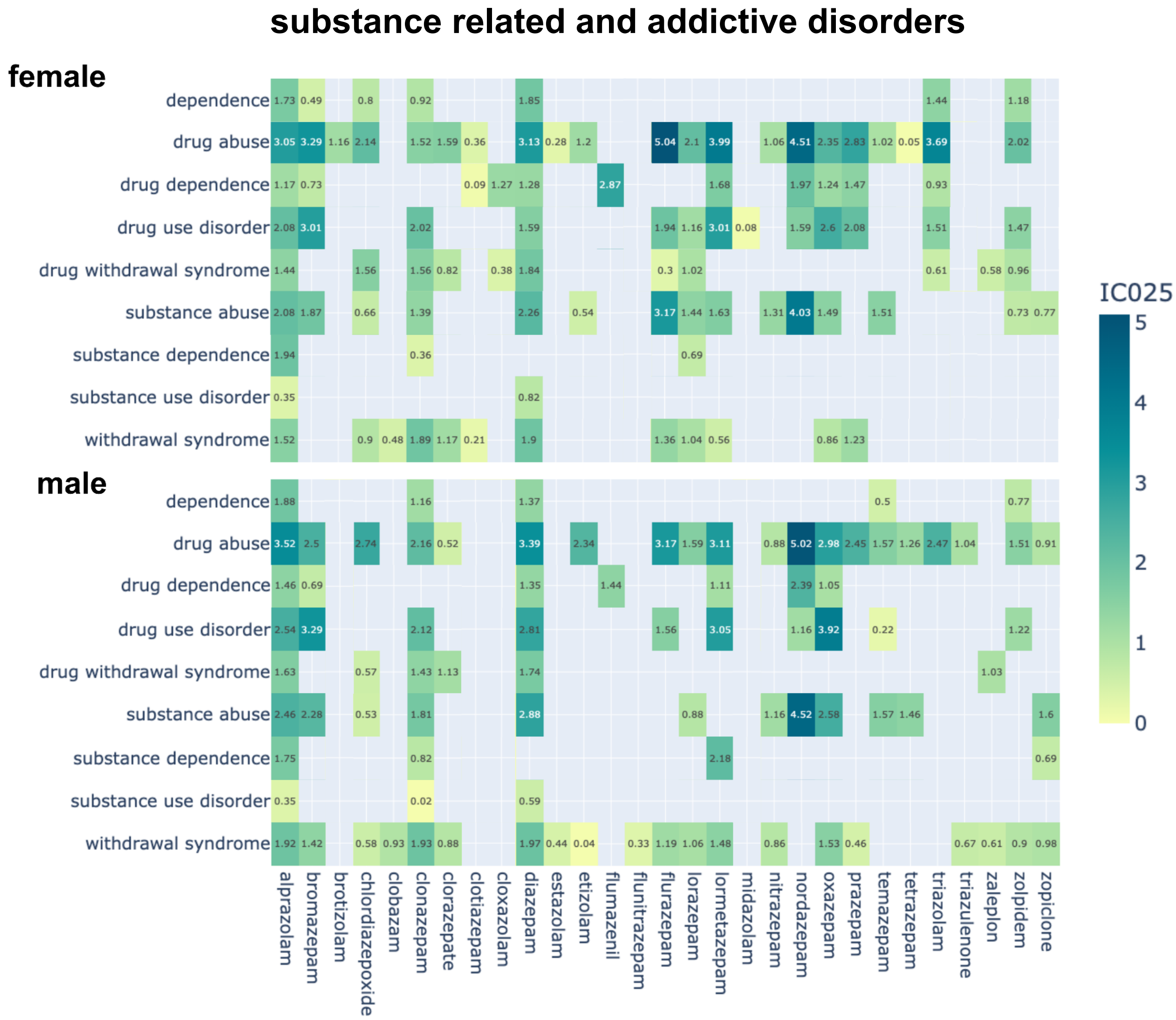

Supplementary Figure 8: *FAERS signals for signs of compound abuse, compound dependence and withdrawal symptoms: The IC025 per AE and drug is plotted as a heatmap for all results from data pool 2. Of the 29 drugs for which associations are found, the signal strength is very diverse: Flumazenil is the special case of the clinically used antagonist. Brotizolam, clobazam, clotiazepam, cloxazolam, and zaleplon are only very weakly associated with signs of abuse and/ or dependence, and some of these lack associations with withdrawal symptoms. This is fully consistent with anecdotal evidence for highly diverse withdrawal severity for different compounds. Alprazolam and diazepam, both being highly prescribed and also broadly available as illicit substances, are*

associated with the whole spectrum. Interestingly, flurazepam and nordazepam have the strongest signals for abuse despite the low prescription rates for nordazepam.

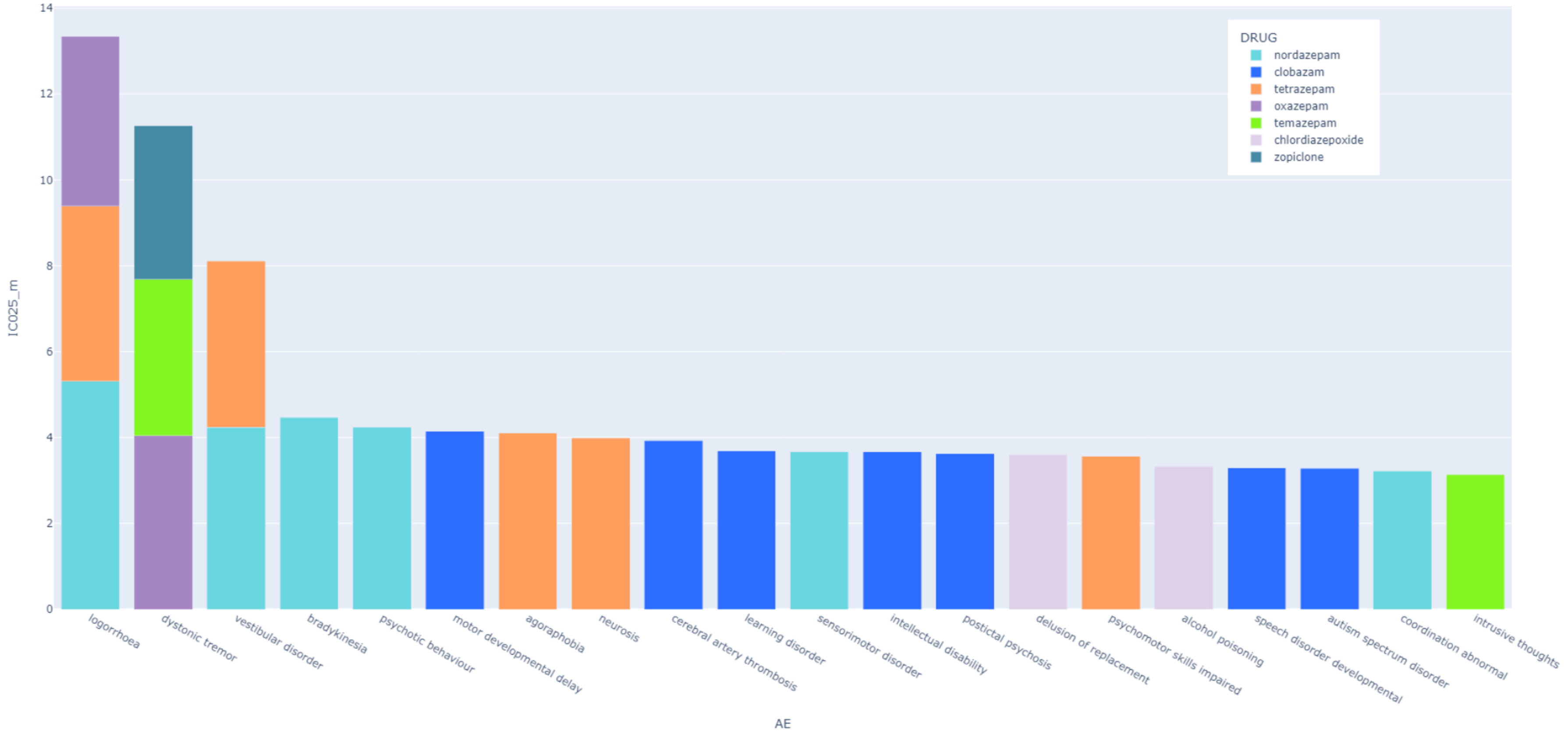

Supplementary Figure 9: Data from Figure 9A: *The 20 AEs from Figure 8 which have the highest IC025 in males and lack positive drug-AE association in females. This is data from the y-axis in Fig 8.*

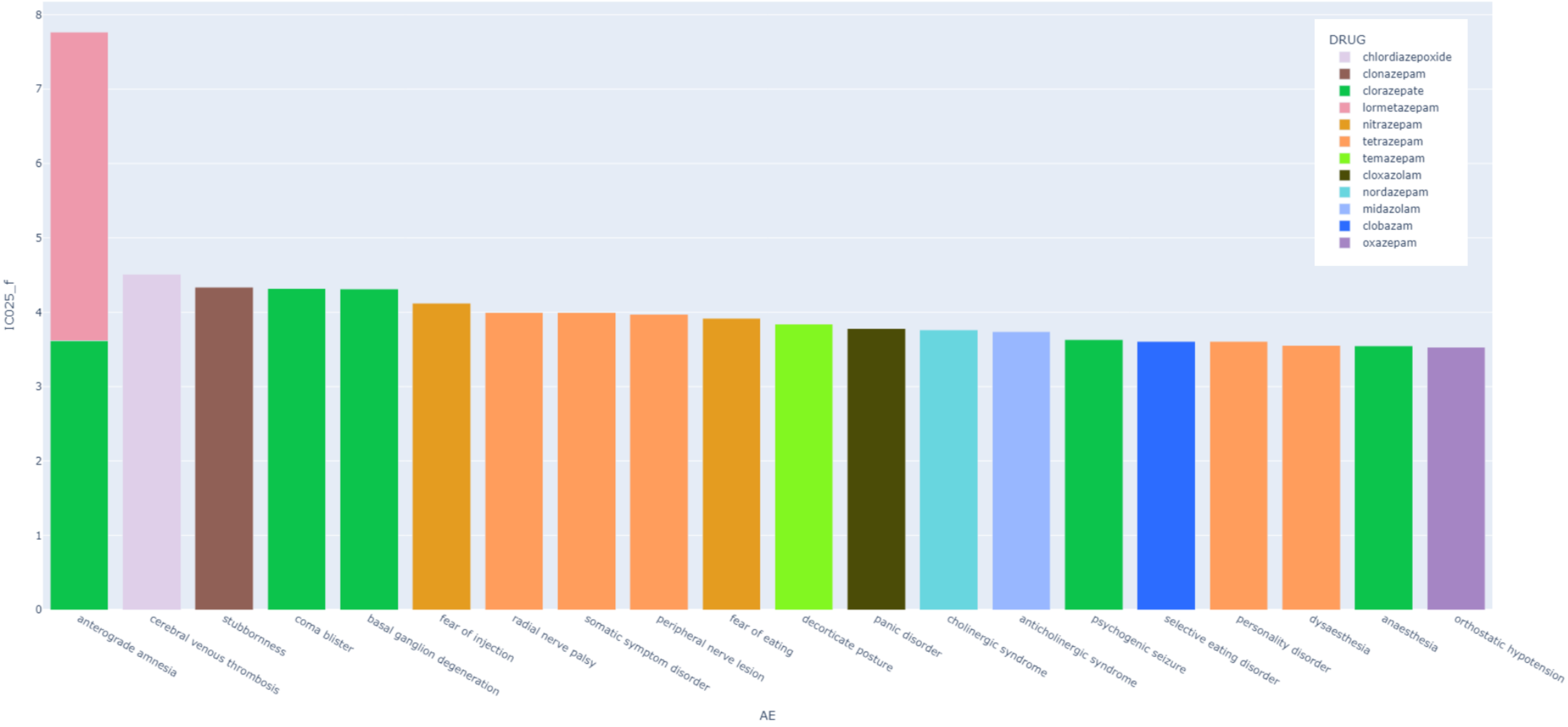

Supplementary Figure 10 Data from Figure 9A: *The 20 AEs from Figure 8 which have the highest IC025 in females and lack positive drug-AE association in males. This is data from the x-axis of Figure 8.*

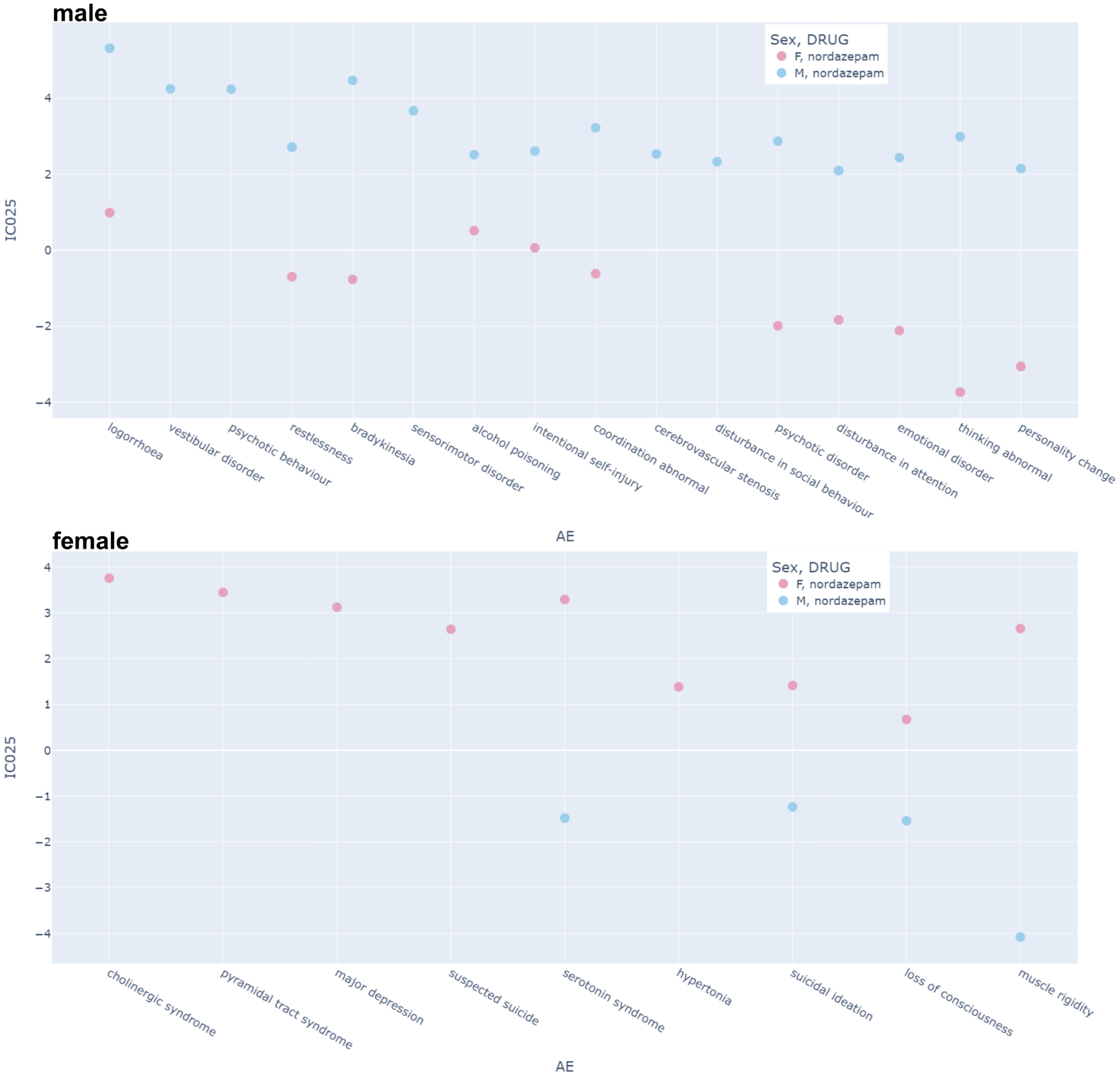

Supplementary Figure 11: *The 20 drug-AE associations (IC025 >0, PRR>2, report number >5) of nordazepam which only occur in one sex and which correlate to the highest IC025 within the neuropsychiatric SOCs are shown for both sexes individually in descending order. The Drug-AE combinations for the other sex (pool 1, not meeting the criteria for an association), are equally shown in a different color; red: female; blue: male. Note that for female only 9 drug-AE associations which do not occur in males are found.*

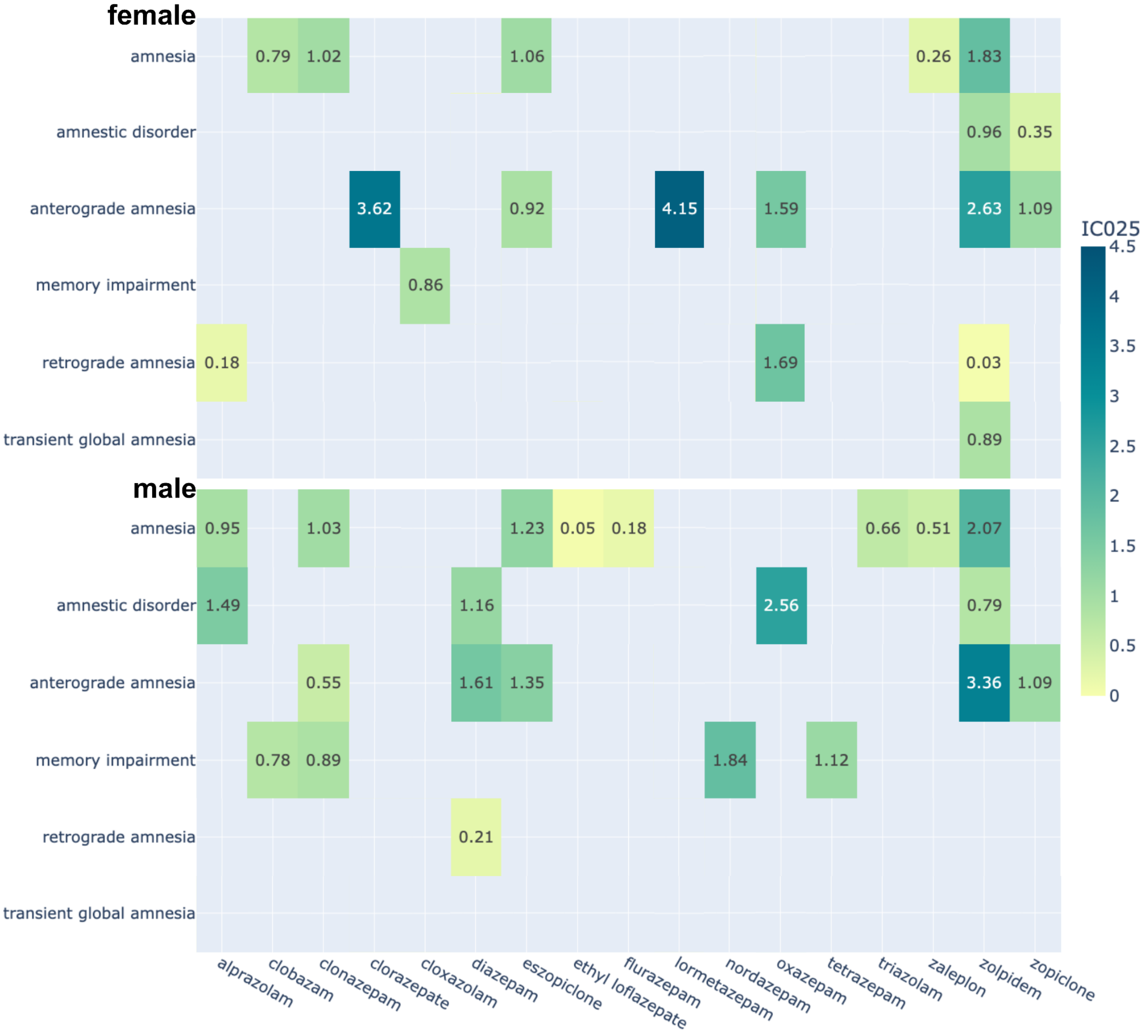

Supplementary Figure 12: *Drugs which have been found to be associated with amnestic symptoms are represented with their IC025 values separated for each sex.*

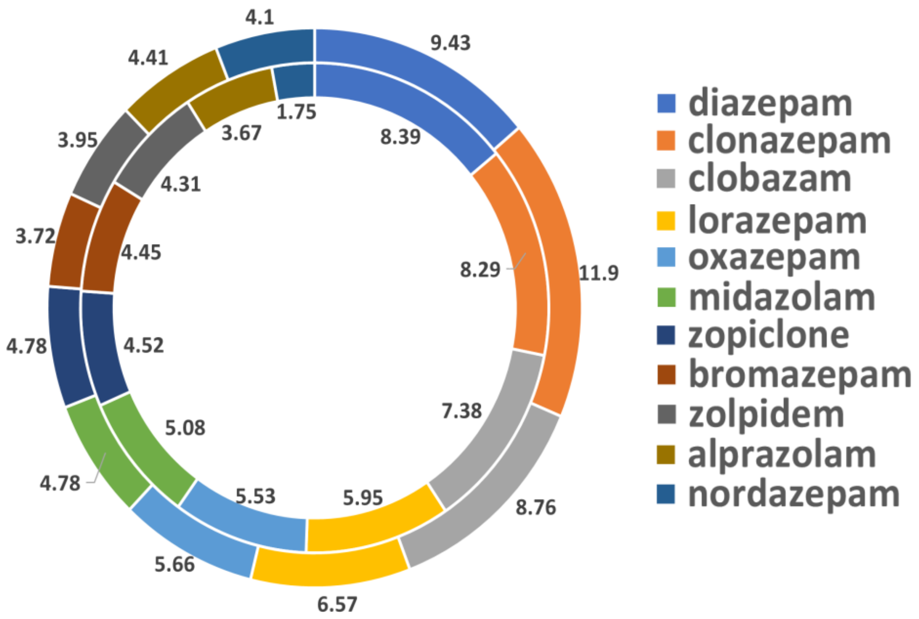

Supplementary Figure 13: *Pie chart presents for the top ten drugs for each sex (together ten or more) with the highest cIC025 contribution to the "nervous system disorders" and "psychiatric disorders" system organ classes (SOCs) in terms of summed cIC025 (ScI025). The size of the displayed segments corresponds to the cIC025 contribution of each drug to the summed cumulative IC025 (ScIC025) and is shown as percentage. The outer circles reflect data for males, while the inner circles represent data for females, with the drugs sorted according to the female ScI025 rank values, starting at the top in clockwise direction descending.*

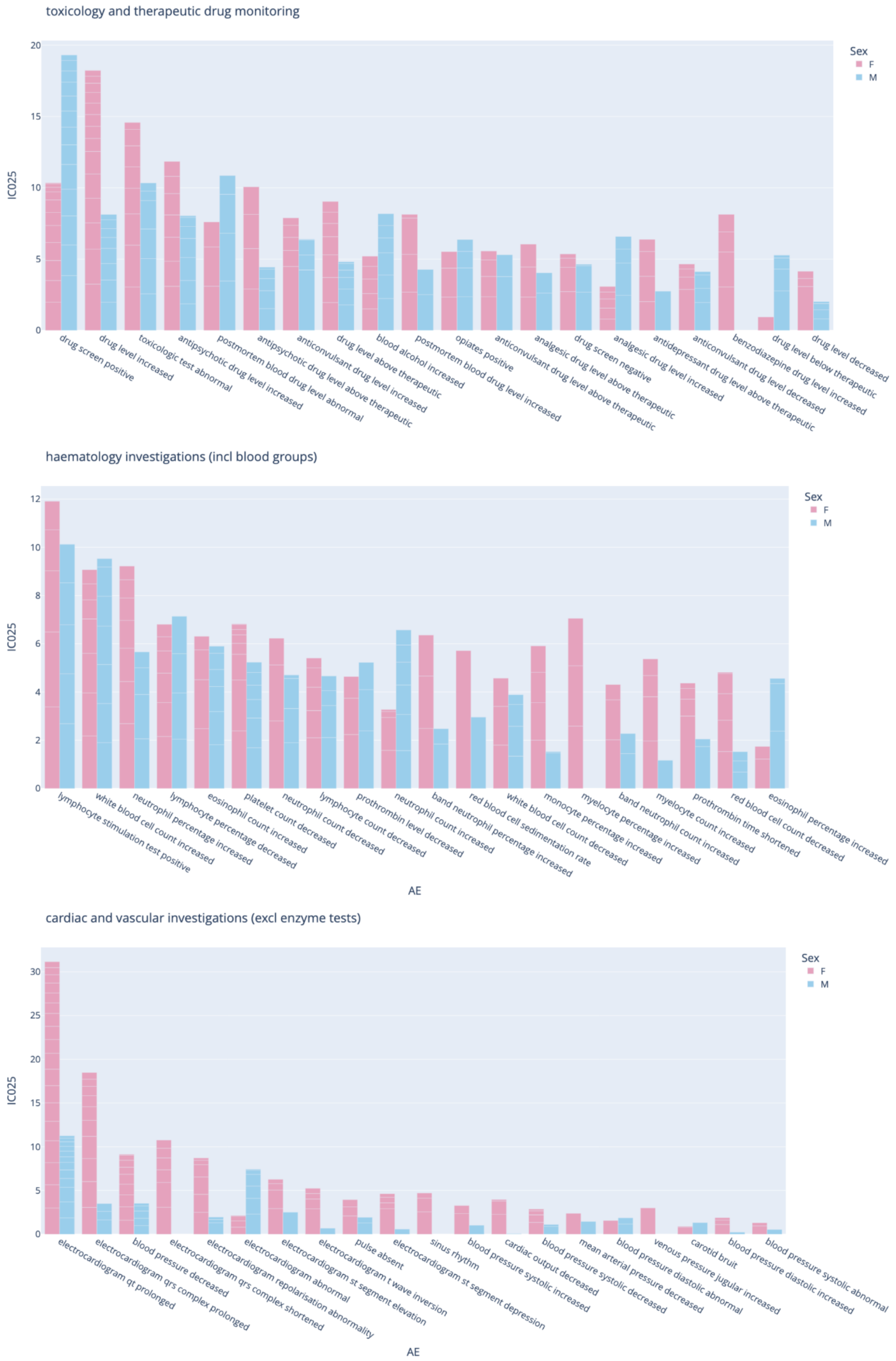

Supplementary Figure 14: *20 AEs with the highest cumulative IC025 in descending order within the HLGs of the SOC "investigations", that show the highest cIC025 for both sexes.*

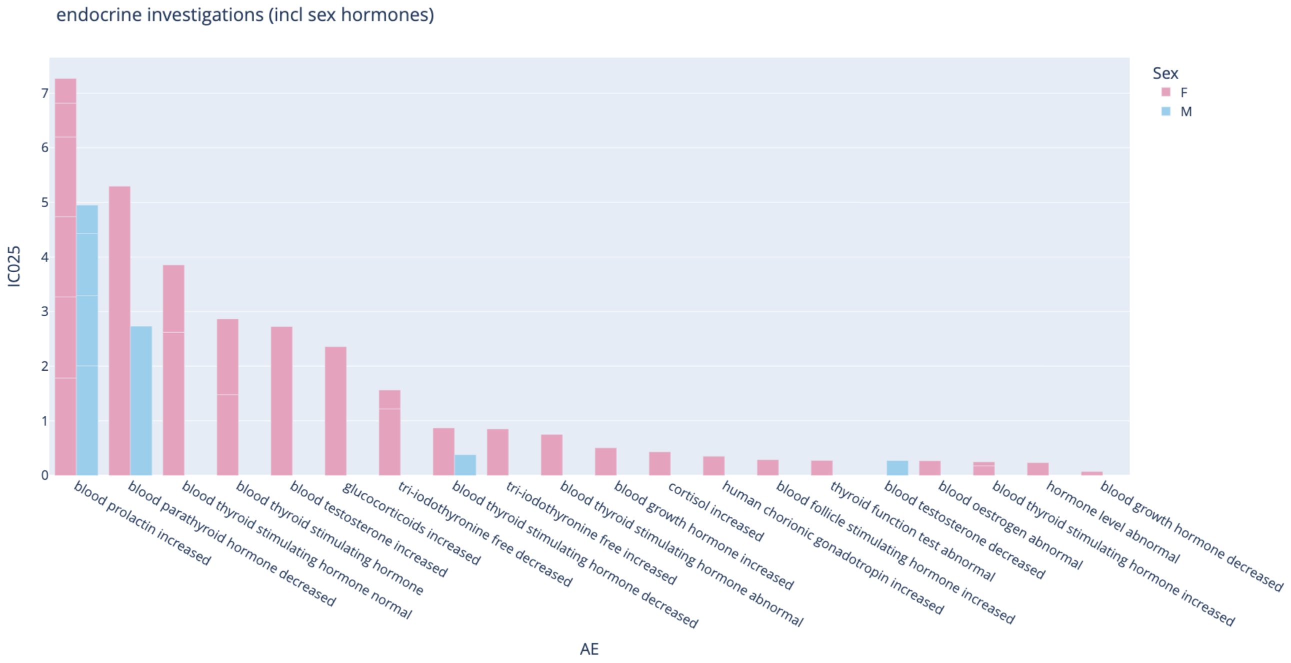

Supplementary Figure 15: *The cumulative IC025 values for the most prevalent 20 adverse events related to endocrine investigations (including sex hormones), for each sex separately.*

2.2 Supplementary Tables

Table S1: *List of benzodiazepines and Z-drugs that were integrated into the manuscript before and after filtering. Note that the after filtering list was applied to further analysis such as ligand fingerprints and 2D-ligand feature plots (MS fig X, Y)*

drugs having FAERS records	after disproportionate analysis (female)	after disproportionate analysis (male)
alprazolam	alprazolam	alprazolam
bentazepam		
bromazepam	bromazepam	bromazepam
brotizolam	brotizolam	brotizolam
chlordiazepoxide	chlordiazepoxide	chlordiazepoxide
cinolazepam	cinolazepam	cinolazepam
clobazam	clobazam	clobazam
clonazepam	clonazepam	clonazepam
clorazepate	clorazepate	clorazepate
clotiazepam	clotiazepam	clotiazepam
cloxazolam	cloxazolam	cloxazolam
diazepam	diazepam	diazepam
estazolam	estazolam	estazolam
eszopiclone	eszopiclone	eszopiclone
ethyl loflazepate	ethyl loflazepate	ethyl loflazepate
etizolam	etizolam	etizolam

flumazenil	flumazenil	flumazenil
flunitrazepam	flunitrazepam	flunitrazepam
flurazepam	flurazepam	flurazepam
halazepam		
ketazolam	ketazolam	ketazolam
lorazepam	lorazepam	lorazepam
lormetazepam	lormetazepam	lormetazepam
medazepam		medazepam
mexazolam	mexazolam	
midazolam	midazolam	midazolam
nimetazepam		
nitrazepam	nitrazepam	nimetazepam
nordazepam	nordazepam	nitrazepam
oxazepam	oxazepam	nordazepam
oxazolam	oxazolam	oxazepam
pinazepam		
prazepam	prazepam	prazepam
quazepam	quazepam	quazepam
remimazolam		
temazepam	temazepam	temazepam
tetrazepam	tetrazepam	tetrazepam
tofisopam	tofisopam	
triazolam	triazolam	triazolam
triazulenone	triazulenone	triazulenone

zaleplon	zaleplon	zaleplon
zolazepam		
zolpidem	zolpidem	zolpidem
zopiclone	zopiclone	zopiclone

Table 2: *The highest five ranked cIC025s for the top four HLGs in the psychiatric disorder SOC supporting data which was used in figure 7A. The drugs within the five highest ranks, which overlap between both sexes are coloured in yellow.*

Drug	Sex	cIC025 [rank]	HLG
diazepam	female	23.69 [1]	psychiatric disorders nec
alprazolam	female	20.62 [2]	psychiatric disorders nec
clonazepam	female	20.36 [3]	psychiatric disorders nec
flurazepam	female	18.08 [4]	psychiatric disorders nec
lorazepam	female	17.97 [5]	psychiatric disorders nec
diazepam	male	30.59 [1]	psychiatric disorders nec
oxazepam	male	23.90 [2]	psychiatric disorders nec
clonazepam	male	22.89 [3]	psychiatric disorders nec
alprazolam	male	22.24 [4]	psychiatric disorders nec

nordazepam	male	19.84 [5]	psychiatric disorders nec
eszopiclone	female	33.90 [1]	sleep disorders and disturbances (incl subtypes)
zolpidem	female	31.15 [2]	sleep disorders and disturbances (incl subtypes)
clonazepam	female	21.31 [3]	sleep disorders and disturbances (incl subtypes)
zaleplon	female	11.67 [4]	sleep disorders and disturbances (incl subtypes)
diazepam	female	9.46 [5]	sleep disorders and disturbances (incl subtypes)
zolpidem	male	32.75 [1]	sleep disorders and disturbances (incl subtypes)
eszopiclone	male	32.64 [2]	sleep disorders and disturbances (incl subtypes)
clonazepam	male	26.50 [3]	sleep disorders and disturbances (incl subtypes)
diazepam	male	14.34 [4]	sleep disorders and disturbances (incl subtypes)
clobazam	male	10.72 [5]	sleep disorders and disturbances (incl subtypes)
diazepam	female	14.59 [1]	suicidal and self-injurious behaviours nec

flurazepam	female	11.98 [2]	suicidal and self-injurious behaviours nec
clonazepam	female	11.22 [3]	suicidal and self-injurious behaviours nec
oxazepam	female	10.36 [4]	suicidal and self-injurious behaviours nec
lorazepam	female	10.21 [5]	suicidal and self-injurious behaviours nec
diazepam	male	15.53 [1]	suicidal and self-injurious behaviours nec
alprazolam	male	10.70 [2]	suicidal and self-injurious behaviours nec
clonazepam	male	10.47 [3]	suicidal and self-injurious behaviours nec
flurazepam	male	9.82 [4]	suicidal and self-injurious behaviours nec
zopiclone	male	9.41 [5]	suicidal and self-injurious behaviours nec
clonazepam	female	28.95 [1]	anxiety disorders and symptoms
alprazolam	female	20.51 [2]	anxiety disorders and symptoms
diazepam	female	18.63 [3]	anxiety disorders and symptoms

zopiclone	female	16.17 [4]	anxiety disorders and symptoms
lorazepam	female	12.57 [5]	anxiety disorders and symptoms
clonazepam	male	31.16 [1]	anxiety disorders and symptoms
diazepam	male	22.79 [2]	anxiety disorders and symptoms
alprazolam	male	20.33 [3]	anxiety disorders and symptoms
zolpidem	male	13.24 [4]	anxiety disorders and symptoms
tetrazepam	male	13.07 [5]	anxiety disorders and symptoms
